# A Spatial Model of Hepatic Calcium Signaling and Glucose Metabolism Under Autonomic Control Reveals Functional Consequences of Varying Liver Innervation Patterns Across Species

**DOI:** 10.3389/fphys.2021.748962

**Published:** 2021-11-26

**Authors:** Aalap Verma, Alexandra Manchel, Rahul Narayanan, Jan B. Hoek, Babatunde A. Ogunnaike, Rajanikanth Vadigepalli

**Affiliations:** ^1^Department of Biomedical Engineering, University of Delaware, Newark, DE, United States; ^2^Daniel Baugh Institute for Functional Genomics and Computational Biology, Department of Pathology, Anatomy, and Cell Biology, Thomas Jefferson University, Philadelphia, PA, United States; ^3^Department of Chemical and Biomolecular Engineering, University of Delaware, Newark, DE, United States

**Keywords:** calcium dynamics, liver lobule, glucose metabolism, innervation, computational modeling, spatial calcium patterns, cell-cell interactions, cross-species

## Abstract

Rapid breakdown of hepatic glycogen stores into glucose plays an important role during intense physical exercise to maintain systemic euglycemia. Hepatic glycogenolysis is governed by several different liver-intrinsic and systemic factors such as hepatic zonation, circulating catecholamines, hepatocellular calcium signaling, hepatic neuroanatomy, and the central nervous system (CNS). Of the factors regulating hepatic glycogenolysis, the extent of lobular innervation varies significantly between humans and rodents. While rodents display very few autonomic nerve terminals in the liver, nearly every hepatic layer in the human liver receives neural input. In the present study, we developed a multi-scale, multi-organ model of hepatic metabolism incorporating liver zonation, lobular scale calcium signaling, hepatic innervation, and direct and peripheral organ-mediated communication between the liver and the CNS. We evaluated the effect of each of these governing factors on the total hepatic glucose output and zonal glycogenolytic patterns within liver lobules during simulated physical exercise. Our simulations revealed that direct neuronal stimulation of the liver and an increase in circulating catecholamines increases hepatic glucose output mediated by mobilization of intracellular calcium stores and lobular scale calcium waves. Comparing simulated glycogenolysis between human-like and rodent-like hepatic innervation patterns (extensive vs. minimal) suggested that propagation of calcium transients across liver lobules acts as a compensatory mechanism to improve hepatic glucose output in sparsely innervated livers. Interestingly, our simulations suggested that catecholamine-driven glycogenolysis is reduced under portal hypertension. However, increased innervation coupled with strong intercellular communication can improve the total hepatic glucose output under portal hypertension. In summary, our modeling and simulation study reveals a complex interplay of intercellular and multi-organ interactions that can lead to differing calcium dynamics and spatial distributions of glycogenolysis at the lobular scale in the liver.

## Introduction

The liver plays a critical role in mammalian physiology by regulating a wide range of metabolic processes including the balance of blood glucose levels. With its ability to control glucose uptake from and release into circulating blood by tuning the activity of appropriate enzymes, the liver is able to modulate glycogenolysis, gluconeogenesis, glycogenesis, and glycolysis – the major pathways involved in glucose metabolism in the liver – thereby maintaining short and longer-term glucose homeostasis (Nordlie et al., 1999; Radziuk and Pye, 2001; Wahren and Ekberg, 2007; Han et al., 2016). Disruption of glucose metabolism in the liver can lead to chronic disease as is well**-**known in the association of non-alcoholic fatty liver disease (NAFLD) with metabolic syndrome and type II diabetes (Jiang and Zhang, 2003; Parekh and Anania, 2007; Chao et al., 2019).

The mammalian liver serves as a reservoir of glycogen, which can be broken down by hepatocytes into glucose and released into the circulating blood. Hepatic glycogenolysis is primarily regulated by glucagon secreted by the pancreas. In response to increased demand of circulating glucose, such as during exercise, synthesis and secretion of glucagon increases by autonomic activation and signaling through the splanchnic nerve. Increased circulating glucagon induces rapid glycogenolysis in the liver. In addition to glucagon, glycogenolysis in the liver can be modulated by catecholamines during periods of starvation or intense physical activity (Chu et al., 1996). Release of epinephrine into the circulating blood supply is driven by the central nervous system (CNS) sensing of blood glucose levels and subsequent autonomic signaling to the adrenal glands through the activation of splanchnic nerve fibers (Livingstone et al., 1995; Burcelin et al., 2000; Marty et al., 2007; Oosterveer and Kristina, 2014). Activation of adrenergic receptors on hepatocytes by circulating epinephrine leads to glycogenolysis-inducing downstream signaling (Sherwin and Sacca, 1984). Additionally, activation of the adrenergic receptors elicits calcium spiking within hepatocytes through a Gq protein coupled receptor signaling pathway (Orellana et al., 1987; Baukal et al., 1988; Wolf et al., 1988; Gaspers et al., 2019). Interestingly, glycogenolysis is a calcium-dependent process. An increase in cytosolic calcium can enhance the rate of glycogenolysis through the activation of glycogen phosphorylase kinase (Assimacopoulos-Jeannet et al., 1977; Billah and Michell, 1979). Glycogenolysis can be affected similarly by the release of norepinephrine at the autonomic nerve endings within the liver, representing a direct mode of CNS control of hepatic glucose metabolism (Exton, 1987). Catecholamines can thus initiate glucose release and simultaneously accelerate its production to facilitate quick response of the liver to organismal metabolic demands.

Although all hepatocytes can break down glycogen, there is heterogeneity in the extent of glycogenolysis within hepatocytes (Jungermann, 1983). This heterogeneity is driven by a combination of several factors. The liver consists of repeating hexagonal units, termed lobules, each of which is organized around a central vein. Vertices of the hexagonal lobules are occupied by portal triads, containing the portal vein, bile duct, and hepatic artery. Hepatocytes are aligned along sinusoids that connect the portal region of lobules to the central vein. Blood flows through the sinusoids from the periportal to the pericentral region. The directionality of blood flow through the lobule creates a natural gradient of circulating hormonal stimulus to hepatocytes lined along the sinusoids, which translates into varying levels of activation of intracellular signaling and metabolic processes (Jungermann and Keitzmann, 1996). The inherent zonation of hepatocellular metabolic processes and cell-intrinsic calcium signaling regulators contributes to the heterogeneity of glycogenolytic activity of hepatocytes. Expression of glycogenolytic genes has been shown to be more active in the periportal region of liver lobules, with a progressive decrease toward the pericentral region (Jungermann, 1988; Jungermann and Kietzmann, 2000). In contrast, calcium “waves” in the liver lobule, driven by intercellular exchange of inositol 1,4,5-trisphosphate (IP_3_), travel from the pericentral zone to the periportal zone (Amaya and Nathanson, 2013). Such intercellular calcium “waves” are responsible for integrating hormonal control of glucose output in the intact liver, as demonstrated by Gaspers et al. (2019). The opposing gradients of cell intrinsic calcium signaling parameters and circulating hormones can combine to influence zonal contributions toward total glucose output within lobules.

**Figure 1:**
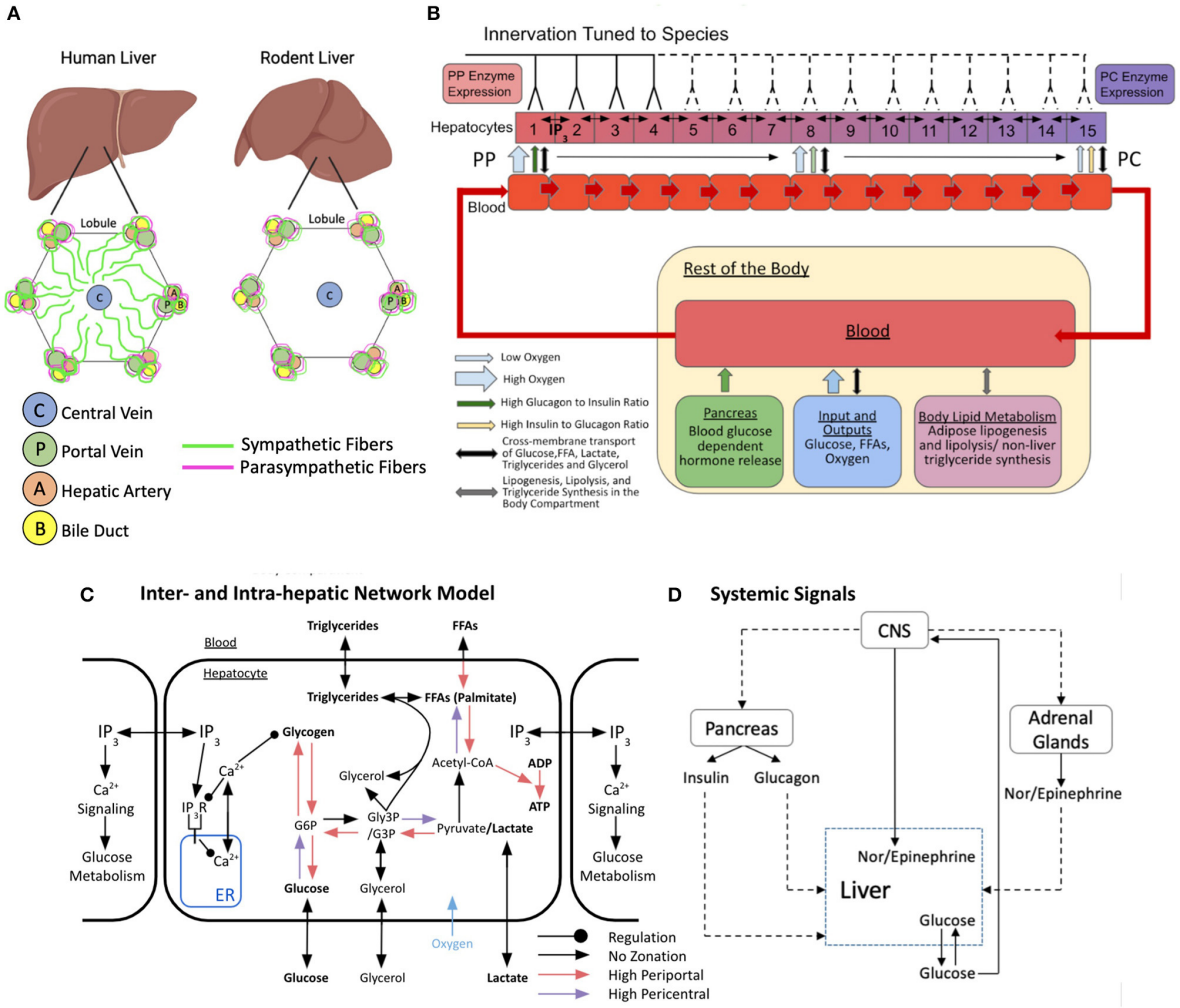
Schematic representation of the multi-organ model of glucose metabolism and calcium signaling under autonomic control. **(A)** Hepatic innervation patterns across species. **(B)** Lobular scale model with varying degrees of hepatic innervation tuned to species. The lobule is represented as a repeating chain of hepatocytes and sinusoidal blood compartments that interact via cross-membrane transport. Hepatocytes can freely exchange IP3 with adjacent neighbors. Blood progresses from hepatic compartment 1–15 and exits into a larger compartment representing the rest of the body. The systemic blood compartment interacts with the pancreas, adrenal glands, adipose tissue, and central nervous system (CNS). **(C)** Inter and intra-hepatic network model with zonated intracellular processes and regulation. **(D)** Multi-organ interaction network of systemic signals. Figures B and C were adapted and modified from Ashworth et al. (2016).

An additional layer of hepatic glycogenolysis control is provided by hepatic innervation. The liver is innervated by both sympathetic fibers from the splanchnic nerve and parasympathetic nerve fibers under the control of the vagus nerve (Berthoud, 2004; McCuskey, 2004; Yi et al., 2010; Jensen et al., 2013; Mizuno and Ueno, 2016). Interestingly, the extent of hepatic innervation varies significantly across species. At the liver lobule scale, very few nerve fibers make contact with hepatocytes in rodents, limited only to the periportal region. On the other hand, in humans, nearly every hepatic lobular layer receives neural input (Figure 1A; Reilly et al., 1978; Metz and Forssmann, 1980; Akiyoshi et al., 1998). As a result of autonomic stimulation, norepinephrine can be released at synapses between hepatic neurons and hepatocytes. Although norepinephrine does not play a significant role in glycogenolysis, it can increase glycogenolysis rates in hepatocytes indirectly by inducing Ca^2+^ oscillations in hepatocytes (Yamaguchi and Garceau, 1980; Jungermann et al., 1987). Innervation patterns of the liver can thus modulate zonal contributions to hepatic glucose synthesis through induction of Ca^2+^ signaling. Yet, it has been shown that both rats and humans promote glycogenolysis through similar mechanisms of activation (Amaya and Nathanson, 2013). This raises an interesting question: How are rodents able to compensate for the sparsity of direct neural input to liver parenchyma? One hypothesis postulates that the extent of hepatic innervation is inversely proportional to the amount of gap junctions across species. This hypothesis stems from the work of Forssmann and Ito (1977) and Reilly et al. (1978) in which they show that the density of gap junctions is low in the tree shrew and the guinea pig but high in rats. Similar to humans, tree shrews and guinea pigs exhibit nerve contacts with nearly all hepatocytes (Forssmann and Ito, 1977; Reilly et al., 1978; Metz and Forssmann, 1980). This potential compensatory relationship between innervation and gap junction connectivity remains unresolved.

Since innervation is crucial for metabolic functionality of the liver, denervation, or loss of function of hepatic nerves can affect liver function adversely causing systemic issues. Functional or physical loss of hepatic innervation can occur due to different reasons. Loss of nerve function was observed in livers of streptozotocin-induced diabetic mice (Stümpel et al., 1996). Cirrhotic livers have been reported to exhibit loss of parenchymal innervation (Lee et al., 1992). Effects of loss of hepatic innervation have been widely studied in the context of insulin sensitivity (Mikines et al., 1985; Xie and Lautt, 1996). However, the effects of hepatic denervation on liver glucose output and the effect due to lobular scale calcium dynamics remains poorly understood.

In the present study, we used a multi-scale modeling approach to explore the dynamic behavior of a complex network of intracellular signaling, intercellular, and multi-organ interactions governing calcium signaling and glycogenolysis in the liver. We developed a multi-scale, multi-organ model of central regulation of hepatic glucose metabolism, in which we incorporated intracellular glucose and glycogen metabolism, lobular scale zonation of these metabolic processes, hepatic innervation, peripheral organ-mediated hormonal signaling, and tissue scale calcium signaling elicited by systemic hormones (Figures 1B–D). We built upon previously published models of hepatic glucose metabolism, intracellular calcium signaling, and lobule-scale calcium dynamics, integrating a new model of direct and indirect control of liver metabolism by the central nervous system through catecholaminergic processes. We evaluated, via simulation, a range of scenarios including species-specific differences in liver innervation, alteration of lobule-scale gap junction connectivity, and disruption of local and global neural signals. Specifically, we examined the impact of these variations on calcium-mediated regulation of liver glucose metabolism and hepatic glucose output.

## Materials and Methods

### Multi-Scale Model Development

Our model builds on the previously developed multi-organ model of hepatic glucose, lipids, and triglyceride metabolism from Ashworth et al. (2016). Briefly, Ashworth et al. (2016) developed an ordinary differential equations (ODE)-based model to simulate and explore zonal features of insulin resistance and hepatic steatosis in non-alcoholic fatty liver disease under periodic feeding-fasting cycles. They modeled metabolic zonation in the liver lobules by treating the porto-central axis of the sinusoid as a repeating unit, with the blood supply and associated hepatocytes split into compartments based on their positions along the lobular axis (Figure 1B). In this scheme, each sinusoidal compartment interacts with local hepatocytes, each of which is affected by hormone signals and exchanges metabolites within its own blood compartment. Blood enters the periportal compartment and flows through to the pericentral compartment where it deposits into a larger body compartment. The original Ashworth et al. (2016) model captures the extensive metabolic and hormone signaling network present in hepatocytes. A detailed description of the original model and the parametric sensitivity analysis can be found in Ashworth et al. (2016). In the present study, we extended the Ashworth et al. (2016) model to include intracellular calcium signaling and intercellular IP3 signaling models into the compartmental model of the liver lobule (Figure 1C). In addition, we included adrenal glands and the central nervous system as part of the body compartments external to the liver (Figure 1D). Our expanded multi-scale model includes additional features and modifications necessary for studying autonomic control of zonated hepatic glucose metabolism, as detailed below.

### Modeling Porto-Central Axis of Human Liver

We expanded the 8-compartment Ashworth et al. (2016) model to consist of 15 compartments within a hepatic lobule to account for the extent of the porto-central axis within a human liver more accurately. In the expanded hepatic lobule model, the first and last four compartments correspond to the periportal and pericentral regions, respectively, and the middle seven compartments correspond to the midlobular region. All 15 compartments exchange molecules with their immediate neighboring blood compartments while the first and fifteenth compartments are in direct contact with systemic blood and exchange molecules within the body compartment. Molecular exchange between all compartments is modeled as mass exchange terms dependent on blood flow rates, according to the following equations:


(1) $$\begin{array}{l} \left(dM_{\left(i\;=\;1:N\right)}\right)/dt=\;bf\cdot \left(M_{\left(i-1\right)}-M_{i}\right) \end{array}$$

(2) $$\begin{array}{l} \left(dM_{0}\right)/dt=\left(bf\;\cdot \;\left(M_{N}\;-\;M_{0}\right)\right)/s \end{array}$$

where *M*_*i*_ denotes the concentration of species *M* in blood compartment *i* (*i* = 1 …. 15), *M*_0_ represents the concentration of species *M* in systemic blood, and *s* represents the body to hepatic compartment ratio: *s* = 5 · *N* (*s* = 75 for the *N* = 15 compartments used). The parameter *bf* represents blood flow through the compartments, which is taken to be *bf* = 0.15·*n s*^−1^ [bf = 2.25*s*^−1^ for the 15 compartments used]. The index *i* = 1 corresponds to the periportal (PP) sinusoidal, and associated hepatocyte compartment, while the index *i* = 15 corresponds to pericentral (PC) compartment.

### Modeling Zonated Metabolic Processes in a Liver Lobule

Molecular exchange between adjacent sinusoidal blood compartments is modeled as mass transfer kinetics. The parameters derived from experimental data of various species were used when considering molecular exchange between a sinusoidal blood compartment and associated hepatocyte. The hepatocyte metabolic network contains both zonated and non-zonated processes consisting of glucose, lipid, fatty acid, and phosphate molecules with their relevant intermediates. Since the oxygen concentration in the sinusoidal blood decreases from the PP to PC region due to uptake by hepatocytes, there exists a gradient of metabolic processes following a specific zonated profile (Jungermann and Kietzmann, 1997). The Ashworth et al. model considers zonation of hepatocyte functions to be dependent on the hepatocyte-specific oxygen concentration along the porto-central axis. The metabolic reactions with higher flux in the periportal zone in which the oxygen concentration is the highest are free fatty acid (FFA) transport between sinusoidal blood compartment and hepatocyte, conversion of FFA into palmitate, ADP to ATP conversion, pyruvate to glucose-3-phosphate (G3P) conversion, G3P to glucose-6-phosphate (G6P) conversion, G6P to glucose conversion, G6P to glycogen conversion, and glycogen to G6P conversion. The metabolic reactions with higher flux in the pericentral zone in which the oxygen concentration is the lowest are glucose to G6P conversion, G3P to pyruvate conversion, and Acetyl-Co-A to FFA conversion (Figure 1C).

### Modeling Systemic Insulin and Glucagon Secretion

The model accounts for systemic insulin and glucagon secretion by the pancreas. Both hormones are metabolized during their passage through the sinusoidal compartments from PP to PC, leading to a zonated concentration profile. Rates of glucagon and insulin secretion in the model are dependent on systemic glucose concentration and the systemic glucose reference value (*G*_*ref*_). If the systemic glucose concentration is less than the reference value, glucagon is secreted and if the systemic glucose concentration is greater than the reference value, insulin is secreted. The rate of glucagon (*Glu*) and insulin (*Ins*) secretion are given by the following equations and the details of parameterization can be found in Ashworth et al. (2016):


(3) $$\begin{array}{l} dGlu/dt=1/\tau_{glu}\;\cdot \text{ln}{\left(G_{ref}/G_{B}\right)}^{\left(n_{glu}\right)}/[{\left(k_{m}^{glu}\right)}^{\left(n_{glu}\right)}\\ +\text{ ln}{\left(G_{ref}/G_{B}\right)}^{\left(n_{glu}\right)}]\;,\;if\;G_{B}\;<\;G_{ref} \end{array}$$

(4) $$\begin{array}{l} dIns/dt=1/\tau_{ins}\;\cdot \text{ln}{\left(G_{ref}/G_{B}\right)}^{\left(n_{ins}\right)}/[{\left(k_{m}^{ins}\right)}^{\left(n_{ins}\right)}\\ +\text{ ln}{\left(G_{ref}/G_{B}\right)}^{\left(n_{ins}\right)}]\;,\;if\;G_{B}\;>\;G_{ref} \end{array}$$

where *G*_*ref*_ = 2 *a*.*u*., τ_*glu*_ = 1.3 *s*, $k_{m}^{glu}=1.3nM$, *n*_*glu*_ = 2, τ_*ins*_ = 0.9 *s*, $k_{m}^{ins}=0.9nM$, *n*_*ins*_ = 2.

Both triglyceride synthesis and lipolysis from the gut and adipose tissue are modeled as single lumped equations with rates modeled as Hill-like equations. Additionally, the model includes inputs of blood glucose and free fatty acids (FFAs) to simulate a daily feeding cycle. However, we assume that there is no external blood glucose or FFA input, for example, through dietary intake during the course of simulation.

### Modeling CNS Activation by Systemic Glucose

We expanded the above model to include autonomic control of zonated hepatic glucose metabolism. Activation of the CNS was a key component in the expanded model, to account for central autonomic control of multiple organs for coordinating the response to a systemic glucose demand. CNS activation was modeled using systemic glucose-dependent saturation kinetics, given by the following equation:


(5) $$\begin{array}{l} CNSAct\;=\;\left(G_{symp}\;-\;G_{B}\right)/(K_{m}^{symp}\\ +\;\left(G_{symp}\;-\;G_{B}\right)),\;if\;G_{B}\;<\;G_{symp} \end{array}$$

where $K_{m}^{symp}=\;500\;\mu M$ and *G*_*symp*_ = 4500 μ*M*.

The formulation considers CNS activation as occurring only when blood glucose levels decrease below 4,500 μ*M*, as normal blood glucose levels range between 3,500 and 5,500 μ*M* (Güemes et al., 2015). For simplicity, we assume that blood glucose sensing occurs only at the hypothalamus, which orchestrates all peripheral hormonal signals and metabolic processes (Livingstone et al., 1995; Marty et al., 2007).

### Modeling Calcium Regulation of Glucose Homeostasis

Calcium dynamics have been shown to regulate glucose dynamics in hepatocytes, as an increase in Ca^2+^ results in an increase in glucose output and glycogenolysis (Billah and Michell, 1979). This regulatory effect of Ca^2+^ is mediated by the activation of glycogen phosphorylase kinase (Assimacopoulos-Jeannet et al., 1977). Our model assumes basal activation of glycogen phosphorylase kinase and a minimum additional cytosolic Ca^2+^ amplitude-dependent activation threshold (*CaI*^*GPK*^ = 0.44 μ*M*). We account for the subsequent glycogen phosphorylase activation implicitly in the glycogenolysis as follows:


(6) $$\begin{array}{l} v_{i}^{GPK}=\{\begin{array}{l} \frac{CaI_{i}}{K_{CaI}^{GPK}+\;CaI_{i}};\;CaI_{i}\;>\;CaI^{GPK}\\ v_{0}\;;\;CaI_{i}\;<\;CaI^{GPK} \end{array} \end{array}$$

(7) $$\begin{array}{l} dGlyc/dt=\;-(v_{brk}\cdot K_{Phos}^{max}\\ \cdot {\left(Glyc\right)}^{\left(n_{brk}\right)})/({\left(Glyc\right)}^{\left(n_{brk}\right)}\\ +\;{\left(K_{m}^{Glyc}\right)}^{\left(n_{brk}\right)})\cdot Phos/(K_{m}^{Phos}\\ +\;Phos)\cdot \left(1\;+\;v_{CaI}^{max}\cdot v_{i}^{GPK}\right) \end{array}$$

(8) $$\begin{array}{l} K_{Phos}^{max}=\left(Glu\;+\;k_{LP}\right)/\left(Ins\;+\;k_{IP}\right) \end{array}$$

where $K_{CaI}^{GPK}=3\;\mu M$, *v*_0_ = 0.05, $K_{m}^{Glyc}=100\;mM$, $K_{m}^{Phos}=4000\;\mu M$, *n*_*brk*_ = 4, $v_{brk}=5\;\mu M\cdot s^{-1}$, *k*_*LP*_ = 45 *pM*, *k*_*IP*_ = 26.66 *pM*. $v_{CaI}^{max}=3$ is the maximum increase in glycogen phosphorylase induced by Ca^2+^, with a Michaelis-Menten constant of $K_{m}^{CaI}=0.6\;\mu M$. *Phos* represents the intracellular phosphate concentration. The glycogen phosphorylase kinase activity parameter is a dimensionless quantity that modulates the rate of glycogenolysis within each hepatocyte.

### Modeling Calcium Signaling Across the Porto-Central Axis

The present expanded model couples the intra-hepatocyte metabolic model developed by Ashworth et al. (2016) with a Ca^2+^ spiking model (Verma et al., 2016) based on the IP_3_-Ca^2+^ cross coupling proposed by Meyer and Stryer (1991). Briefly, the Ca^2+^ spiking model accounts for extracellular stimulus-induced activation of the intracellular Ca^2+^ signaling cascade. Ca^2+^ spiking occurs due to the positive feedback by IP_3_-induced Ca^2+^ release and Ca^2+^-stimulated IP_3_ formation. We model the induction of Ca^2+^ signals by extracellular catecholamine (epinephrine and norepinephrine) stimulus through α_1_-adrenergic receptor activation, however, similar Ca^2+^ dynamics can be induced by other extracellular stimuli such as vasopressin (Assimacopoulos-Jeannet et al., 1977; Schöfl et al., 1993; Verma et al., 2018). The equations for intracellular Ca^2+^ fluxes and IP_3_ receptor dynamics are retained from Meyer and Stryer (1991) with the exception of IP_3_ synthesis, which is related to the hormone receptor dynamics instead of constant receptor activation in the original Meyer and Stryer (1991) model.

The present multicellular model of Ca^2+^ signal propagation includes gap junction-mediated intracellular communication for a chain of *N* connected hepatocytes. In Equations 9-13 below, the subscript *i* represents the cell index for hepatocyte specific species and parameters. For each adjacent cell pair, IP_3_ exchange is modeled by a mass exchange term with the mass transfer parameter *G*_*ij*_. Although Ca^2+^ can normally pass through the gap junctions, for the simplicity of our model and the lower diffusion constant of Ca^2+^ compared with IP_3_ (560 vs. 280 um^2^/s respectively), the model only assumes IP_3_ exchange between adjacent hepatocytes. Additionally, considering the short intracellular diffusion time compared with typical spike temporal width (~30 s) and inter-spike intervals (~100 s), the IP_3_ concentration is assumed to be uniform throughout the cytosol. We approximate IP_3_ exchange between adjacent hepatocytes as a mass exchange term owing to the small latency period compared with typical spike widths and inter-spike intervals.

Binding of extracellular catecholamines to cognate receptors on the cell membrane leads to activation of the intracellular Ca^2+^ signaling cascade. While receptor internalization is possible, the model assumes that the total receptor number remains constant. The Ca^2+^ spiking state of every hepatocyte is characterized by four different variables: cytosolic Ca^2+^ concentration (*CaI*), cytosolic IP_3_ (*IP*3), ratio of free (unbound) to total IP_3_ receptors (*g*) on the endoplasmic reticulum surface, and the ratio of free (unbound) to total stimulus receptors on the cell surface (*r*). In Equation 9, *R*_*f*_ denotes the cell surface density of free α_1_-adrenergic receptors, whereas *R*_*total*_ represents the total cell surface density of α_1_-adrenergic receptors. The rate of increase in free receptor density is assumed to be proportional to the number of bound receptors, with rate constant *k*_*r*_. The density of free receptors is assumed to decrease through two different modes of binding: non-specific binding of α_1_-adrenergic receptors is represented by *k*_*d*_ · *R*_*f*_ and binding of free α_1_-adrenergic receptors to catecholamines is assumed to follow mass action kinetics with rate constant *k*_*Hr*_. Dividing Equation 9 by *R*_*total*_ yields the following equation for the balance of free α_1_-adrenergic receptor fraction, as shown in Equation 10. Here, *r* represents the fraction of free (unbound) α_1_-adrenergic receptors on the cell surface.


(9) $$\begin{array}{l} \left(dR_{\left(f,\;i\right)}\right)/dt=k_{ri}\cdot \left(R_{total}-R_{\left(f,\;i\right)}\right)\\ -k_{d}\cdot R_{\left(f,\;i\right)}\;-\;k_{Hr}\cdot H_{i}\cdot R_{\left(f,\;i\right)} \end{array}$$

(10) $$\begin{array}{l} \left(dr_{i}\right)/dt=k_{\left(r_{i}\right)}\cdot \left(1-r_{i}\right)\;-k_{d}\cdot r_{i}\\ -\;k_{Hr}\cdot H_{i}\cdot r_{i} \end{array}$$

where $k_{\left(r_{i}\right)}\in \left[0.5,\;1\right]\;s^{-1}$, $k_{d}=0.34\;s^{-1}$, $k_{Hr}=1\;nM^{-1}\cdot s^{-1}$. Parameterization of *H*_*i*_ (*nM*) is described in further detail in Equations (16) and (17).

We assume saturation kinetics for catecholamine induced IP_3_ synthesis in the cytosol as expressed in Equation (11). The positive feedback between cytosolic Ca^2+^ (*CaI*) and IP_3_ is expressed as a multiplicative first order Hill function. Metabolism of IP_3_ in the cytosolic domain can occur through two different routes: conversion into IP_4_ by a 3-kinase and conversion into IP_2_, IP_1_, and, eventually, inositol by a phosphatase (Sims and Allbritton, 1998). In the current model, the rate of IP_3_ decrease is assumed to be single mass action decay, which is expressed as follows:


(11) $$\begin{array}{l} \left(dIP3_{i}\right)/dt=\left(k_{\left(IP3_{i}\;\right)}\cdot H\cdot r_{i}\right)/(k_{cat}\\ \;+\;r_{i}\;)\cdot \left(CaI_{i}\right)/\left(CaI_{i}\;+\;k_{3}\;\right)-D\cdot IP3_{i}\\ \;-G_{ij}\cdot \left(IP3_{i}-IP3_{j}\right) \end{array}$$

where $k_{\left(IP3_{i}\right)}\in \left[0.5,\;0.6\right]\;s^{-1}$, $G_{ij}=\left\{0,\;2.5,\;5\right\}\;s^{-1}$, *k*_*cat*_ = 0.45, *k*_3_ = 1 μ*M*, *D* = 0.8 *s*^−1^.

The cytosolic Ca^2+^ concentration is modeled such that the rate of increase in cytosolic Ca^2+^ depends on the fraction of bound IP_3_R (1 − *g*). Additionally, there is positive feedback coupling between IP_3_ and cytosolic Ca^2+^. The parameter *L* represents constitutive leakage from the cellular Ca^2+^ stores into the cytosol, while *CaT* represents the concentration of Ca^2+^ in the cellular stores. A second order Hill function describes the initiation of active transport mechanisms pumping Ca^2+^ back into ER stores at the expense of ATP, when there is an increase in cytosolic Ca^2+^. This process is modeled as given in Equation 12.


(12) $$\begin{array}{l} \left(dCaI_{i}\right)/dt=\left(1-g\right)\cdot (\left(A\cdot IP3_{i}^{4}\right)/\left({\left(k_{1}\;+\;IP3_{i\;}\right)}^{4}\;\right)\\ \;+L)\cdot \left(CaT_{i}-CaI_{i}\right)-\left(B\cdot CaI_{i}^{2}\right)/(k_{2}^{2}\\ \;+\;CaI_{i}^{2}\;) \end{array}$$

where *A* = 0.20 μ*M* · *s*^−1^, *k*_1_ = 1 μ*M*, *L* = 0.00015 μ*M* · *s*^−1^, *B* = 0.082 μ*M* · *s*^−1^, *k*_2_ = 0.15 μ*M*.

The balance of free (unbound) to total IP_3_ receptors is represented by Equation 13, where *g* is the fraction of free (unbound) IP_3_R on the ER surface. The rate of inactivation of IP_3_R by cytosolic Ca^2+^, *E*, is proportional to the fourth power of cytosolic Ca^2+^ concentration. The model also assumes a constitutive reactivation rate of IP_3_R, denoted by *F*.


(13) $$\begin{array}{l} \left(dg_{i}\right)/dt=E\cdot CaI_{i}^{4}\cdot \left(1-g_{i}\right)-F \end{array}$$

where *E* = 1 μ*M*^−4^ · *s*^−1^, *F* = 0.01 *s*^−1^.

Detailed description of parameters and initial conditions for the lobular scale Ca^2+^ portion of the model can be found in Tables 1, 2, respectively. We explicitly consider the zonation profiles of the following parameters: α_1_-adrenergic receptors exhibit a decreasing PC to PP gradient (Halpern et al., 2017; Verma et al., 2018). Therefore, the intracellular Ca^2+^ signaling parameters *k*_*r*_ and *k*_*IP*3_ are initialized as decreasing gradients from the PC to PP region (*k*_*r*_ ∈ [0.5, 0.6]; *k*_*IP*3_ ∈ [0.5, 1]). The parameter gradients across the 15 hepatic layers can be found in Table 3.

**Table 1:** Nominal values/ranges used to initialize hepatocyte intracellular parameters in the main model of lobular scale Ca^2+^ dynamics.

**Symbol**	**Value**	**Description**
*A*	0.20 μM s^−1^	Maximal rate of Ca^2+^ release from ER store
*B*	0.082 μM s^−1^	Maximal rate of cytosolic Ca^2+^ pump to ER
*D*	0.8 s^−1^	IP3 degradation rate
*E*	1 μM^−4^ s^−1^	IP3R inactivation rate
*F*	0.01 s^−1^	IP3R activation rate
*G*_*ij*_	{0, 2.5, 5} s^−1^	Gap junction mediated IP3 mass transfer coefficient
*k*_1_	1 μM	IP3 concentration for half maximal rate of catalysis of store Ca^2+^ release
*k*_2_	0.15 μM	Cytosolic Ca^2+^ concentration for half maximal pump rate
*k*_3_	1 μM	Cytosolic Ca^2+^ concentration for half maximal rate of IP3 production
*k*_*cat*_	0.45	Bound receptor ratio for half-maximal IP3 production rate
*k*_*d*_	0.34 s^−1^	Hormone independent agonist receptor binding rate
*k*_*Hr*_	1 nM^−1^ s^−1^	Hormone-receptor binding rate
*k*_*IP*3_	[0.5, 0.6] s^−1^	Saturation IP3 synthesis rate (graded along the lobule)
*k*_*r*_	[0.5, 1] s^−1^	Agonist receptor recycling rate (graded along the lobule)
*L*	0.00015 μM s^−1^	Ca^2+^ leakage flux from store to cytosol
*K*_(*NEpn*_*stim*_)_	{0, 2} nM	Direct norepinephrine stimulus to liver

**Table 2:** Initial conditions for all hepatocytes in the model of lobular scale Ca^2+^ dynamics.

**Symbol**	**Value**	**Description**
*CaI*_0_	0.2 μM	Cytosolic Ca^2+^ concentration
*CaT*_0_	500.2 μM	Total Ca^2+^ concentration
*g*_0_	0.25	Ratio of free to total IP3R
*IP*3_0_	0.1 nM	IP3 concentration
*r*_0_	0.5	Ratio of free to total agonist receptors in cell *i*

**Table 3:** *k*_*r*_ and *k*_*IP*3_ parameter gradients along the lobule applicable to the main model.

	***k*_*r*_ (s^−1^)**	***k*_*IP*3_ (s^−1^)**
Hepatocyte 1 (PP)	0.500	0.500
Hepatocyte 2	0.536	0.507
Hepatocyte 3	0.571	0.514
Hepatocyte 4	0.607	0.521
Hepatocyte 5	0.643	0.529
Hepatocyte 6	0.679	0.536
Hepatocyte 7	0.714	0.543
Hepatocyte 8	0.750	0.550
Hepatocyte 9	0.786	0.557
Hepatocyte 10	0.821	0.564
Hepatocyte 11	0.857	0.571
Hepatocyte 12	0.893	0.579
Hepatocyte 13	0.929	0.586
Hepatocyte 14	0.964	0.593
Hepatocyte 15 (PC)	1.000	0.600

### Modeling CNS Control of Circulating Catecholamines

Circulating catecholamines play an important role in systemic glucose homeostasis by inducing Ca^2+^ spiking in hepatocytes leading to increased glycogenolysis and glucose output from the liver. We assume a CNS-mediated activation of adrenal glands leading to release of epinephrine and norepinephrine into the systemic blood supply. We use the following equations for rates of increase in systemic epinephrine and norepinephrine:


(14) $$\begin{array}{l} dEpn/dt=v_{Epn}\;\left(1+X_{Epn}*CNSAct\right) \end{array}$$

(15) $$\begin{array}{l} dNEpn/dt=v_{NEpn}\;\left(1+X_{NEpn}*CNSAct\right) \end{array}$$

where $v_{Epn}=0.5\;nM\cdot s^{-1}$; $v_{NEpn}=1.5\;nM\cdot s^{-1}$; *X*_*Epn*_ = 6; *X*_*NEpn*_ = 14. *X*_*Epn*_ and *X*_*NEpn*_ describe the stimulatory factors due to CNS activation. *v*_*NEpn*_ > *v*_*Epn*_ is in accordance with the observation that norepinephrine levels rise faster and attain higher values during exercise (Christensen et al., 1979; Horton et al., 1998). Catecholamines released in the blood stream then pass through the sinusoidal blood compartments and induce intracellular Ca^2+^ spiking. We assume that catecholamines are cleared only in the liver during their passage through sinusoids following mass action kinetics.

### Modeling Catecholamine-Mediated Activation of Signaling in a Liver Lobule

The zonated, short range Ca^2+^ mobilizing effect of norepinephrine was approximated by additional stimulus only to the innervated hepatocytes as shown below:


(16) $$\begin{array}{l} H_{i}=Epn_{i}+NEpn_{i}+K_{NEpn_{stim}}\cdot CNSAct;\\ i\in \;innervatedhepatocytes \end{array}$$

(17) $$\begin{array}{l} H_{i}=Epn_{i}+NEpn_{i};\;i\in \;non-innervatedhepatocytes \end{array}$$

where *Epn*_*i*_ and *NEpn*_*i*_ represent the concentrations of the catecholamines in the *i*^th^ sinusoidal blood compartment and *H*_*i*_ represents the Ca^2+^ mobilizing stimulus to the *i*^th^ hepatocyte compartment. The value of *K*_*NEpn*_*stim*__ was set to 2 nM in all simulations with innervation.

### Modeling Variations in Blood Flow

Given a normal blood flow rate, hepatic portal venous gas (HVPG), or the blood pressure at the hepatic portal vein, is roughly 5 mmHg. In cases of severe portal hypertension, HVPG may increase up to 3-fold, reaching 15 mm Hg, thereby altering normal blood flow rate (Kumar et al., 2008; Lake-Bakaar, 2015). We used Poiseuille's law as denoted in Equation 18 below to describe the blood pressure differential across a liver sinusoid between PP and PC compartments. The proportional relationship between blood pressure and blood flow rate allows for calculation of blood flow rate during severe portal hypertension.


(18) $$\begin{array}{l} \Delta p\;=\;8\mu LQ/\left(\pi R^{2}\;\right) \end{array}$$

where Δ*p* = blood pressure difference across the lobule, μ = viscosity, *L* = length of blood vessel, *Q* = blood flow rate, *R* = sinusoidal radius. For simplicity, we assume there is no change in viscosity of the blood, sinusoidal length, or diameter due to an increase in portal hypertension. Therefore, increased blood flow during portal hypertension can be simulated by increasing the blood flow parameter (*bf* = 6.75 *s*^−1^ for a 15-compartment lobule).

### Model Calibration and Assumptions

#### Metabolism and Zonation

In the current model we have preserved the dynamics of glucose and glycogen metabolism, zonation of related parameters, and systemic glucagon and insulin dynamics published in the original model. We assume that the increase in glucagon modeled in the original work includes the effect of autonomic activation implicitly.

#### Intracellular and Lobular Scale Ca^2+^ Signaling

Under a hormone stimulus, hepatocytes typically exhibit inter-spike intervals of 100 s (Robb-Gaspers and Thomas, 1995; Verma et al., 2018). Based on this rough estimate of inter-spike interval for catecholamine induced Ca^2+^ spikes in hepatocytes, nominal parameter values and initial conditions used in simulations are listed in Tables 1, 2, respectively. The spatial zonation of hormone receptor expression and IP_3_ synthesis governing calcium dynamics are modeled as graded levels of *k*_*r*_ and *k*_*IP*3_ increasing from the PP to PC region, with corresponding values listed in Table 3.

It must, however, be noted that cytosolic Ca^2+^ spiking exhibits wide ranges of inter-spike intervals, durations, and latency periods due to the complex intracellular Ca^2+^ signaling machinery and cell-to-cell heterogeneity. Using single values for model parameters that describe cytosolic Ca^2+^ spikes does not capture this heterogeneity exhibited by individual cells. Additionally, change in extracellular stimulus strength alters the inter-spike interval.

Upon hormonal stimulation, Ca^2+^ waves have typically been observed to propagate at rates between 15 and 25 μm/s (Robb-Gaspers and Thomas, 1995; Gaspers and Thomas, 2005). We tuned the model parameters to match this approximate Ca^2+^ propagation speed. Additionally, we considered Ca^2+^ oscillations induced only by catecholamines.

#### Effect of Catecholamines on Glycogenolysis

We model the effect of catecholamines on hepatic glycogenolysis as mediated explicitly via the potentiation of intracellular Ca^2+^ signaling and a downstream increase in glycogen phosphorylase kinase activity. We calibrated the exercise-induced increase in systemic glucose, epinephrine, and norepinephrine to reflect typical fold changes in human subjects (Horton et al., 1998).

#### Innervation

We assume different spatial innervation patterns in rodent vs. human livers. In the rodent case, we assume innervation and norepinephrine release at the four hepatocyte layers nearest the PP region. In contrast, in the human case, we assume all hepatocyte layers are innervated.

### Simulation Methodology—Effects of Exercise on Glucose Homeostasis in Liver

We analyzed the simulated model output for different Ca^2+^ mobilizing stimuli and gap junction conductivity profiles during exercise. Exercise was modeled as a constantly elevated rate of glucose and FFA uptake by the muscles over a 1 h interval after an initial 500 s interval without exercise. We assumed a 3.5-times elevated rate of glucose uptake during the simulated period of exercise. We did not consider any food intake during the simulated interval. Our model does not consider the hemodynamic changes induced in the liver by CNS activation (Gardemann et al., 1993).

Although connexins are expressed heterogeneously between the PC and PP regions, we assume uniform gap junction conductivity between all adjacent hepatocyte pairs. The strength of gap junction connectivity on the system was investigated by setting *G*_*ij*_ ∈ {0, 2.5, 5}, where *G*_*ij*_ = 0 represents uncoupled gap junctions, *G*_*ij*_ = 2.5 represents weak gap junction connectivity, and *G*_*ij*_ = 5 represents strong gap junction connectivity. Species-specific autonomic control was simulated by altering the number of hepatic layers that were innervated. In simulating human-like extensive innervation, all hepatic layers were innervated and followed the structure of Equation 16. Rodent-like minimal innervation simulations had innervation of the first four periportal hepatocytes, while the following eleven were denervated. Therefore, the first four layers followed the structure of Equation 16, and the following eleven layers followed the structure of Equation 17. Finally, complete denervation of the liver was simulated by setting parameter *K*_(*NEpn*_*Stim*_)_ = 0 in Equation 16, thereby following the structure of Equation 17 for all hepatic layers.

All simulations were performed using XPPAUT (ver. 8.0, Ermentrout, 2002), which is freely available for download at http://www.math.pitt.edu/~bard/xpp/xpp.html, and an XPP-MATLAB interface (ver. 070626, https://github.com/robclewley/xpp-matlab), available for download from GitHub (https://github.com/Daniel-Baugh-Institute/xpp-matlab). Each simulation ran for ~60 s of wall clock time on a 56-core server with Intel Xeon CPU E5-2697. The model was simulated as a simultaneous system of Ordinary Differential Equations (ODEs).

### Model Alternative

In addition to the main model discussed in this manuscript, we simulated a model alternative. The alternative model differs from the main model in the following aspects: (1) Systemic catecholamine synthesis rates are not influenced by CNS feedback, representing a case of denervation of the adrenal medulla, and (2) CNS feedback has an additive effect on glucagon synthesis, representing a case where alternative pathways of glucagon synthesis are activated in the pancreas due to CNS feedback. This yields an alternate set of equations for the glucagon, epinephrine, and norepinephrine balance to replace above Equations (3), (14), and (15) with the below:


(19) $$\begin{array}{l} dGlu/dt=1/\tau_{glu}\;\cdot {(\text{ln}\left(G_{ref}/G_{B}\right)}^{\left(n_{glu}\right)}\\ /\left[{\left(k_{m}^{gln}\right)}^{\left(n_{glu}\right)}\;+\text{ln}\left({\left(G_{ref}/G_{B}\right)}^{\left(n_{glu}\right)}\right)\right]\\ +\left(v_{glu}\cdot CNSAct\right)/\left(k_{m}^{glu}\;+\;CNSAct\right)\;,\;if\;G_{B}\\ <\;G_{ref} \end{array}$$

(20) $$\begin{array}{l} dEpn/dt=v_{Epn} \end{array}$$

(21) $$\begin{array}{l} dNEpn/dt=v_{NEpn} \end{array}$$

where $v_{glu}=0.1\;nM\;s^{-1}\;“\;and{\;}^{\prime \prime }\;k_{m}^{glu}=0.1$. All other alternative model parameters are equivalent to the main model parameters.

In the alternative implementation of the model, *Gij* values were considered as 1 and 2 for weak and strong gap junction connectivity, respectively. The list of altered parameters is included in Table 4. The intracellular Ca^2+^ signaling parameters *k*_*r*_ and *k*_*IP*3_ are initialized as decreasing gradients from the PC to PP region (*k*_*r*_ ∈ [1, 2]; *k*_*IP*3_ ∈ [1, 2]) in the alternate model as these values led to physiologically relevant results during calibration of the model (Table 5). We discuss additional key differences in parameterization between the two models given the case of human-like innervation in Supplementary Figure 1.

**Table 4:** Nominal values/ranges used to initialize hepatocyte intracellular parameters in the alternative model are the same as the main model, with the exception of:

*G*_*ij*_	{0, 1, 2} s^−1^	Gap junction mediated IP3 mass transfer coefficient
*k*_*IP*3_	[1, 2] s^−1^	Saturation IP3 synthesis rate (graded along the lobule)
*k*_*r*_	[1, 2] s^−1^	Agonist receptor recycling rate (graded along the lobule)
*v*_*glu*_	0.1 nM s^−1^	Maximal rate of systemic glucagon production induced by CNS stimulus
$k_{m}^{glu}$	0.1	Half-maximal stimulus level for systemic glucagon production induced by CNS

**Table 5:** *k*_*r*_ and *k*_*IP*3_ parameter gradients along the lobule applicable to the alternative model.

	***k*_*r*_ (s^−1^)**	***k*_*IP*3_ (s^−1^)**
Hepatocyte 1 (PP)	1	1
Hepatocyte 2	1.0714	1.0714
Hepatocyte 3	1.143	1.143
Hepatocyte 4	1.214	1.214
Hepatocyte 5	1.286	1.286
Hepatocyte 6	1.357	1.357
Hepatocyte 7	1.428	1.428
Hepatocyte 8	1.5	1.5
Hepatocyte 9	1.571	1.571
Hepatocyte 10	1.643	1.643
Hepatocyte 11	1.714	1.714
Hepatocyte 12	1.785	1.785
Hepatocyte 13	1.857	1.857
Hepatocyte 14	1.928	1.928
Hepatocyte 15 (PC)	2	2

### Model Reproducibility and Credible Practice

All simulation results and figures presented in the current work were repeated independently by a member of our research group not involved in the original modeling and simulation study. The reproduced results were in agreement with the results presented here. Parameter values in the code were cross-checked for consistency against the values provided in Tables 1–3 of the manuscript. For the purpose of assessing reproducibility, all model files were downloaded from GitHub (https://github.com/Daniel-Baugh-Institute/SpatialLiverModel; ver. 2, 2021), and are also available as a supplement to the present manuscript. Self-assessment of the Ten Simple Rules for Credible Practice in Modeling and Simulation in Healthcare was performed and is included in the supplement (Supplementary File 3, Erdemir et al., 2020).

## Results

We developed a multi-scale multi-organ model of autonomic control of hepatic glucose metabolism through regulation of systemic, intercellular, and intracellular signaling (Figure 1). Built on previous efforts to account for metabolic zonation and blood flow along porto-central axis within a liver lobule (Ashworth et al., 2016), and intra and intercellular calcium signaling along a liver sinusoid (Verma et al., 2016, 2018), the present model combines these earlier models with a new model of hepatic innervation and regulation of systemic hormonal levels to account for CNS control of organismal scale signaling and hepatic calcium dynamics and glucose metabolism (Figure 1). We simulated the multi-scale model to explore hepatic glucose output and systemic glucose recovery under a range of biological conditions, including increased intensity and duration of exercise, and denervation of the adrenal gland and liver. By accounting explicitly for the extent of neural innervation along a liver sinusoid in the model, we were able to assess the impact of variations in hepatic innervation across species. The simulations and results are discussed in the following sections.

### Modeling Metabolic Response to Synaptic and Hormonal Signals in a Liver With Human-Like Extensive Hepatic Innervation

**Figure 2:**
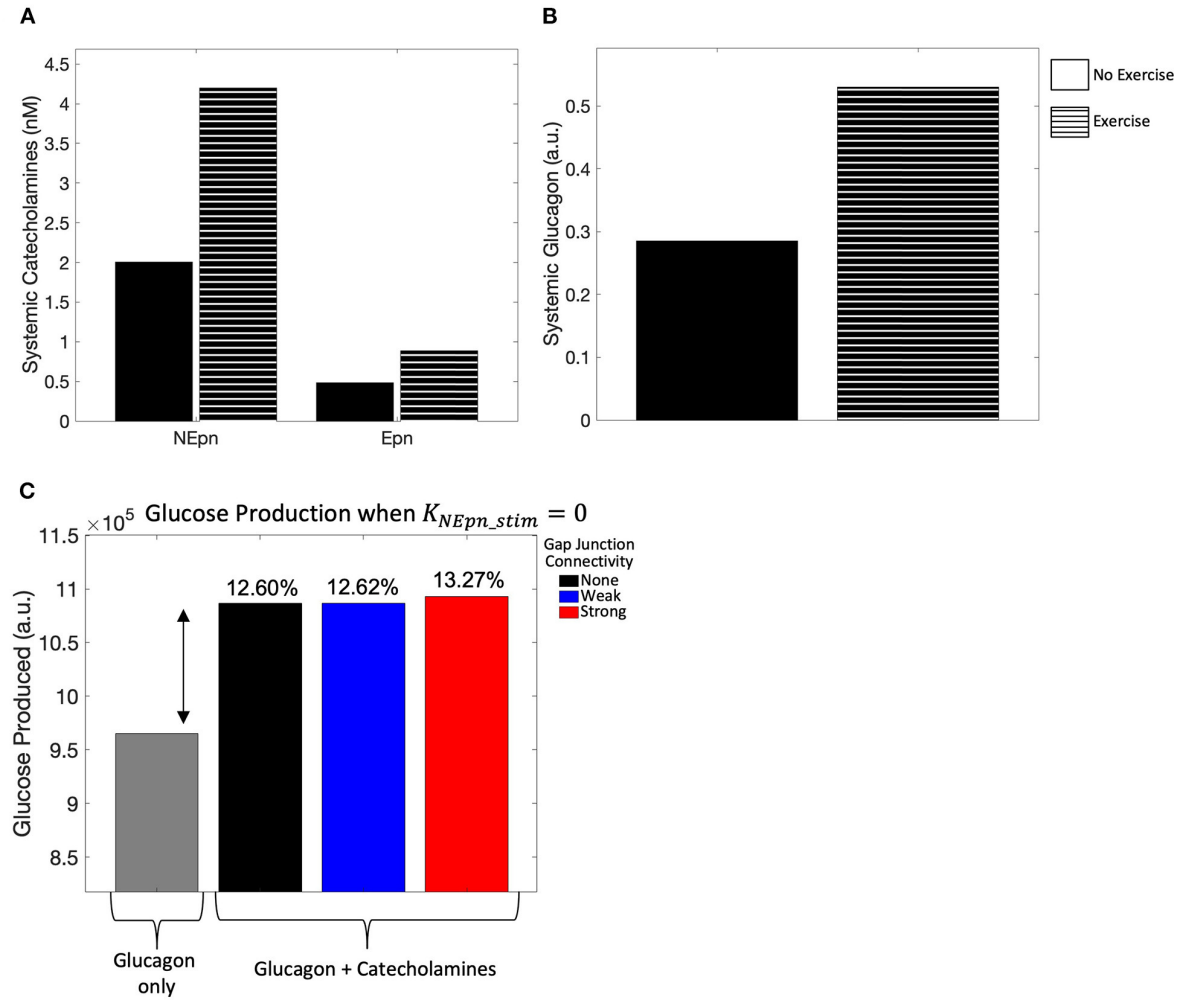
Model calibration. **(A)** Systemic catecholamine concentrations at *t* = 4,000 s, when a pseudo steady-state is reached, show roughly a 2-fold increase with exercise, consistent with the values published in Horton et al. (1998). **(B)** Systemic glucagon concentration at *t* = 4,000 s, when a pseudo steady state is reached, also show roughly a 2-fold increase with exercise. **(C)** Hepatic glycogenolysis only increased by 13.27% for strong gap junction connectivity when systemic catecholamines are released intra-hepatically.

As a qualitative validation of the model, we simulated the effect of no exercise on the model to compare simulated catecholamine and glucagon levels against those during exercise. In agreement with levels reported in Horton et al. (1998), there was roughly a 2-fold increase in systemic epinephrine and norepinephrine concentrations (Figure 2A), while the systemic glucagon concentration (Figure 2B) agreed with the results from the previously developed Ashworth et al. (2016) model. Further validation of the model was conducted by simulating the effect of systemic and locally released hormones on hepatic glycogenolysis. Our simulations showed that hepatic glycogenolysis is primarily driven by glucagon. Systemic catecholamines and norepinephrine released intra-hepatically result in a relatively small increase (13.27% for strong gap junction connectivity) in hepatic glycogenolysis mediated by the initiation of Ca^2+^ response in hepatocytes (Figure 2C).

**Figure 3:**
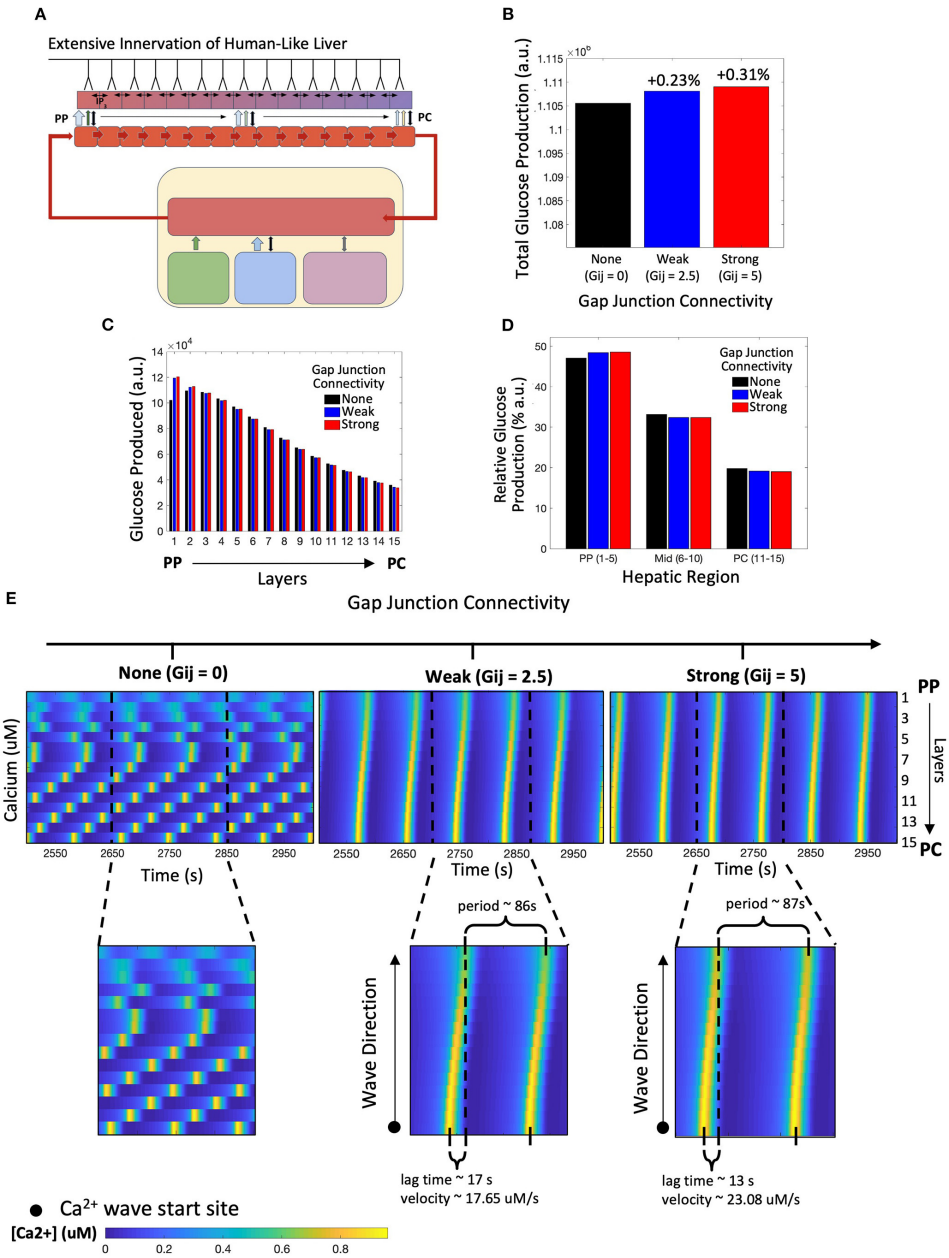
Model of human-like extensive innervation. **(A)** Schematic representation of the model with human-like extensive innervation to all 15 hepatic layers. **(B)** Total hepatic glucose produced increases with strength of gap junction connectivity. **(C)** Hepatic glucose produced across each layer monotonically decreased from the periportal (PP) to pericentral (PC) region for weak and strong gap junction connectivity. **(D)** A small increase in PP glucose output contributes toward the higher total glucose output as gap junction connectivity increases. **(E)** Calcium spiking along the lobule is unsynchronized when there is no gap junction coupling. Weak and strong gap junction connectivity show synchronized calcium wave propagation from the PC to PP region. Calcium wave velocity increases from a system of weak to strong gap junction connectivity.

We simulated the multi-scale multi-organ model in which all layers of the liver lobule received neural inputs, mimicking that of human hepatic innervation (Figure 3A). We considered three distinct scenarios: (1) no gap junctions between hepatocytes, hence no exchange of IP_3_ (*G*_*ij*_ = 0); (2) weak exchange of IP_3_ via gap junctions (*G*_*ij*_ = 2.5); and (3) strong connectivity promoting high IP_3_ flux between adjacent hepatocyte layers (*G*_*ij*_ = 5). Our simulation results show that the total lobular glucose output was not substantially different across the three scenarios, however, a system with strong gap junction connectivity resulted in the largest glucose output, closely followed by a system with weak gap junction connectivity (Figure 3B). Glucose production within the first and second hepatic layers closest to the PP region were most sensitive to variation in gap junction connectivity (Figure 3C). This can be explained by induction of robust intracellular Ca^2+^ response in PP hepatocytes due to gap junction-mediated IP_3_ transfer from adjacent cells. Our simulations further indicated that the periportal zonation of glycogenolysis is preserved in highly innervated livers, as evidenced by the gradient of glucose production shown in Figure 3C. Intrahepatic glucose production was highest in the periportal region, decreasing monotonically toward the pericentral region, irrespective of weak or strong gap junction connectivity (Figure 3C). An increase in gap junction connectivity caused a small increase in the contribution of PP hepatocytes toward total hepatic glucose output (Figure 3D).

These results suggest that when all layers of the liver lobule are innervated, gap junction mediated IP_3_ exchange between hepatocytes has little to no effect on the overall hepatic glucose response to neural and systemic catecholamine signals. This result is consistent with the existing knowledge that hepatic glycogenolysis is regulated primarily by glucagon. However, the intracellular calcium signals elicited in the three scenarios were significantly different. In the simulation with uncoupled hepatocytes, intracellular Ca^2+^ dynamics are unsynchronized, with the Ca^2+^ spikes for each hepatocyte showing its own intrinsic frequency (Figure 3E, left). Gap junction coupling leads to synchronized waves that start in the PC region and propagate toward the PP region, consistent with Ca^2+^ dynamics reported in recent literature (Figure 3E, middle and right; Verma et al., 2018; Gaspers et al., 2019). While the period of Ca^2+^ waves is very similar between the two coupled systems, stronger gap junction connectivity resulted in a shorter lag time (defined as the time between calcium spike maxima of the extreme PC and PP hepatocytes) and a larger Ca^2+^ wave velocity compared with the system with strong gap junction connectivity. However, the system with weak connectivity still displays increased glucose production relative to the uncoupled case, calcium dynamics, and wave velocities similar to those seen experimentally (Robb-Gaspers and Thomas, 1995; Gaspers and Thomas, 2005). Additionally, the amplitude of Ca^2+^ spikes in hepatocytes in the PP region was lower compared with that in mid-lobular and PC regions for both weak and strongly coupled gap junctions (Supplementary Figure 2).

### Modeling Metabolic Response to Synaptic and Hormonal Signals in a Liver With Rodent-Like Minimal Hepatic Innervation

**Figure 4:**
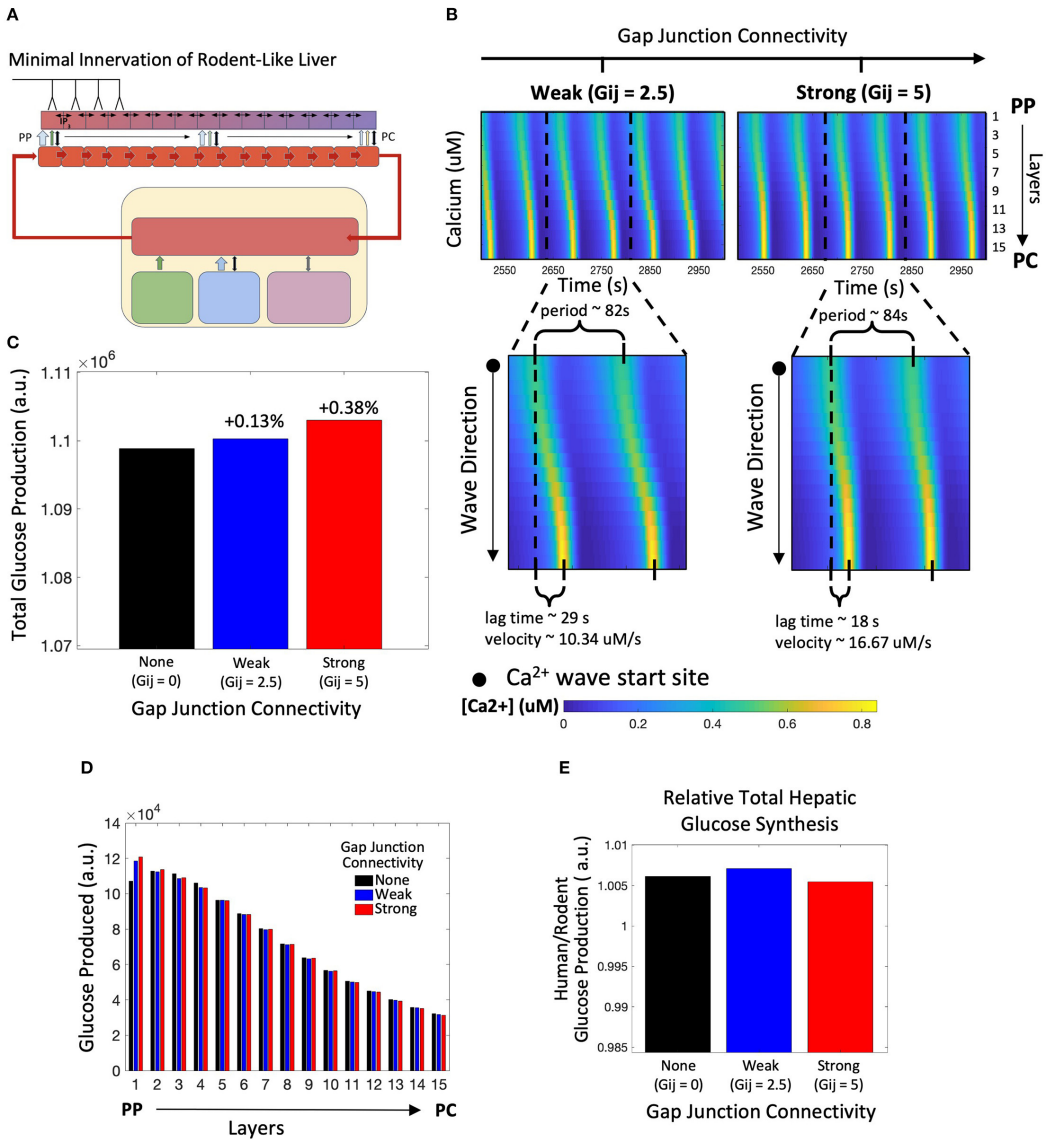
Model of rodent-like minimal innervation. **(A)** Schematic representation of the model with rodent-like minimal innervation to the first 4 periportal layers. **(B)** Calcium waves begin in the PC region despite gap junction connectivity. **(C)** Total hepatic glucose production increases with strength of gap junction connectivity. **(D)** Hepatic glucose production decreases from the PP to PC region with the exception of uncoupled gap junctions. **(E)** Despite gap junction connectivity, there is an increase in total hepatic glucose production from the case of rodent-like minimal innervation to human-like extensive innervation.

Next, we simulated the model innervating only the four hepatocyte layers residing closest to the portal triad (Figure 4A). This scheme of minimal neural inputs in the liver parenchyma serves as a surrogate for the extent of hepatic innervation generally observed in mice and rats (Reilly et al., 1978; Metz and Forssmann, 1980; Akiyoshi et al., 1998). In this case, our simulation results show lobular scale Ca^2+^ dynamics different from those observed following human-like innervation. Ca^2+^ waves in periportally innervated lobules start in the PP region and propagate toward the PC region despite the simulated cell-intrinsic gradients of intracellular Ca^2+^ signaling parameters favoring a PC to PP flow of Ca^2+^ waves (Figure 4B). A combination of direct neuronal stimulus, gradient of circulating catecholamines along the porto-central axis, and spatial gradients of cell-intrinsic Ca^2+^ signaling parameters were responsible for the shift in wave start site between the two cases.

Similar to the case with human-like innervation, our simulations indicate increased total hepatic glucose output with increasing gap junction connectivity (Figure 4C). Despite the PP to PC Ca^2+^ wave across the lobule, the PP zonation of glycogenolysis is preserved (Figure 4D). There is consistently higher hepatic glucose output in the human-like innervation case vs. the rodent-like innervation case (Figure 4E) irrespective of gap junction connectivity due to more innervation and consequently higher catecholamine release at additional neural synapses. Our simulations show that the hepatic glucose output in the case of rodent-like innervation exhibiting strong gap junction connectivity is similar to that of human-like innervation, indicating the compensatory role played by strong gap junction connectivity in poorly innervated livers (Figure 4E).

**Figure 5:**
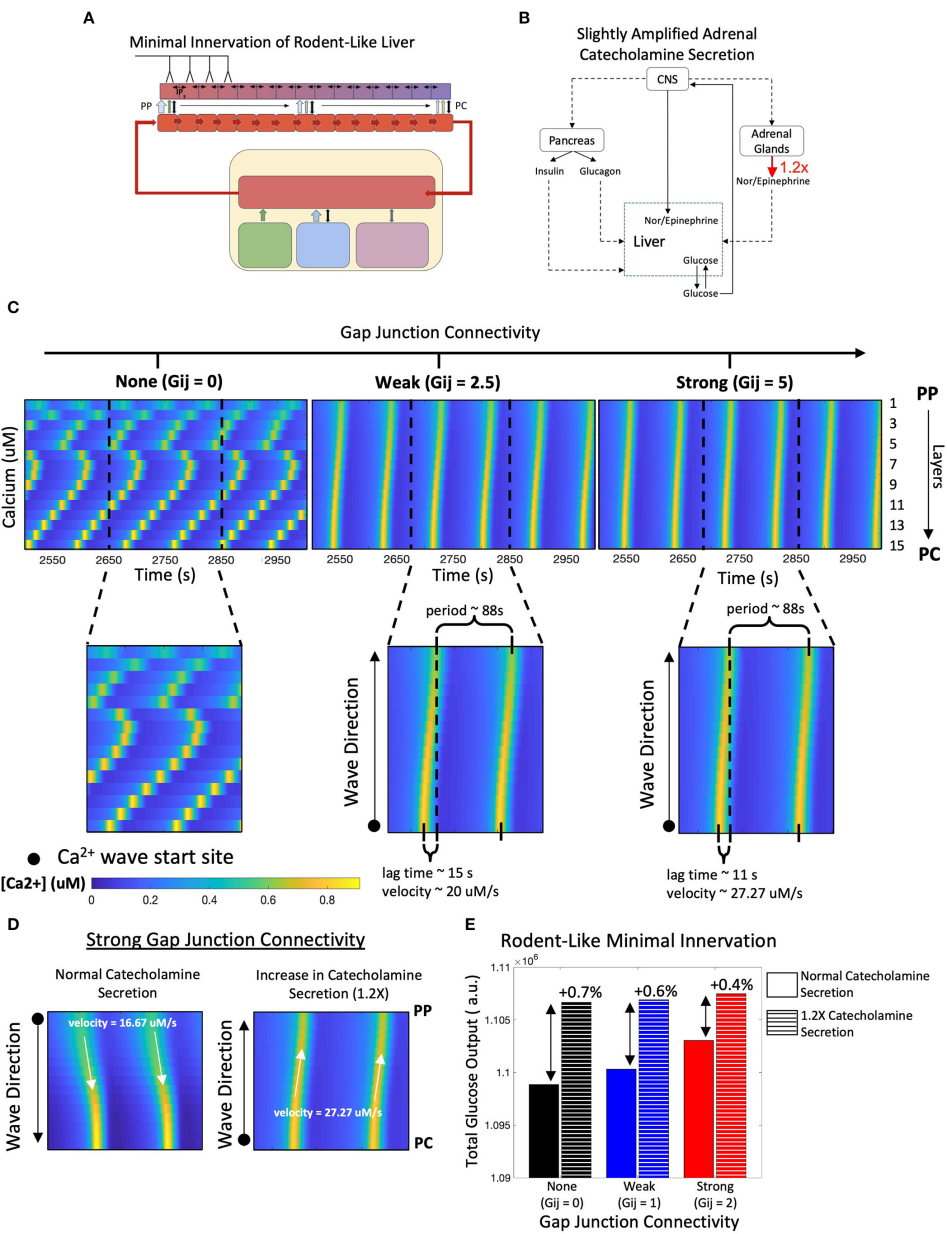
Model of rodent-like minimal innervation with slightly amplified catecholamine secretion (1.2X). **(A)** Schematic representation of model with rodent-like minimal innervation to the first 4 periportal layers. **(B)** Network diagram of systemic signals in which the adrenal glands increase catecholamine secretion by 20%. **(C)** Calcium waves now propagate from the PC to PP regions following a 1.2-fold increase in circulating catecholamine secretion. **(D)** Strong gap junction connectivity in a system with increased catecholamine secretion results in PC to PP calcium waves with higher velocities compared with that of a system with normal catecholamine secretion, in which waves propagate from the PC to PP region with lower velocities. **(E)** Despite gap junction connectivity, there is an increase in total hepatic glucose output from a system of normal catecholamine secretion to one with a 20% increase in catecholamine secretion.

Next, we asked the question: can the PC to PP Ca^2+^ wave propagation in lobules exhibiting minimal innervation be restored by modulating systemic catecholamine levels? To simulate this scenario, we increased catecholamine synthesis rates by adrenal glands by 20% (Figures 5A,B), as baseline circulating catecholamine concentrations are higher in rodents, as reported in de Champlain et al. (1976). Our simulations revealed that with high gap junction connectivity, Ca^2+^ waves travel from PC to PP (Figure 5C). In addition, a higher circulating stimulus led to an increase in Ca^2+^ wave propagation speed and higher hepatic glucose output (Figures 5D,E). Thus, our simulations revealed a compensatory mechanism for poorly innervated livers, mediated by stimulation of extra-hepatic processes, that can mimic Ca^2+^ dynamics and increase total glucose output to levels near those observed in highly innervated livers (Figure 5E).

### Modeling Metabolic Response to Hormonal Signals in a Denervated Liver

**Figure 6:**
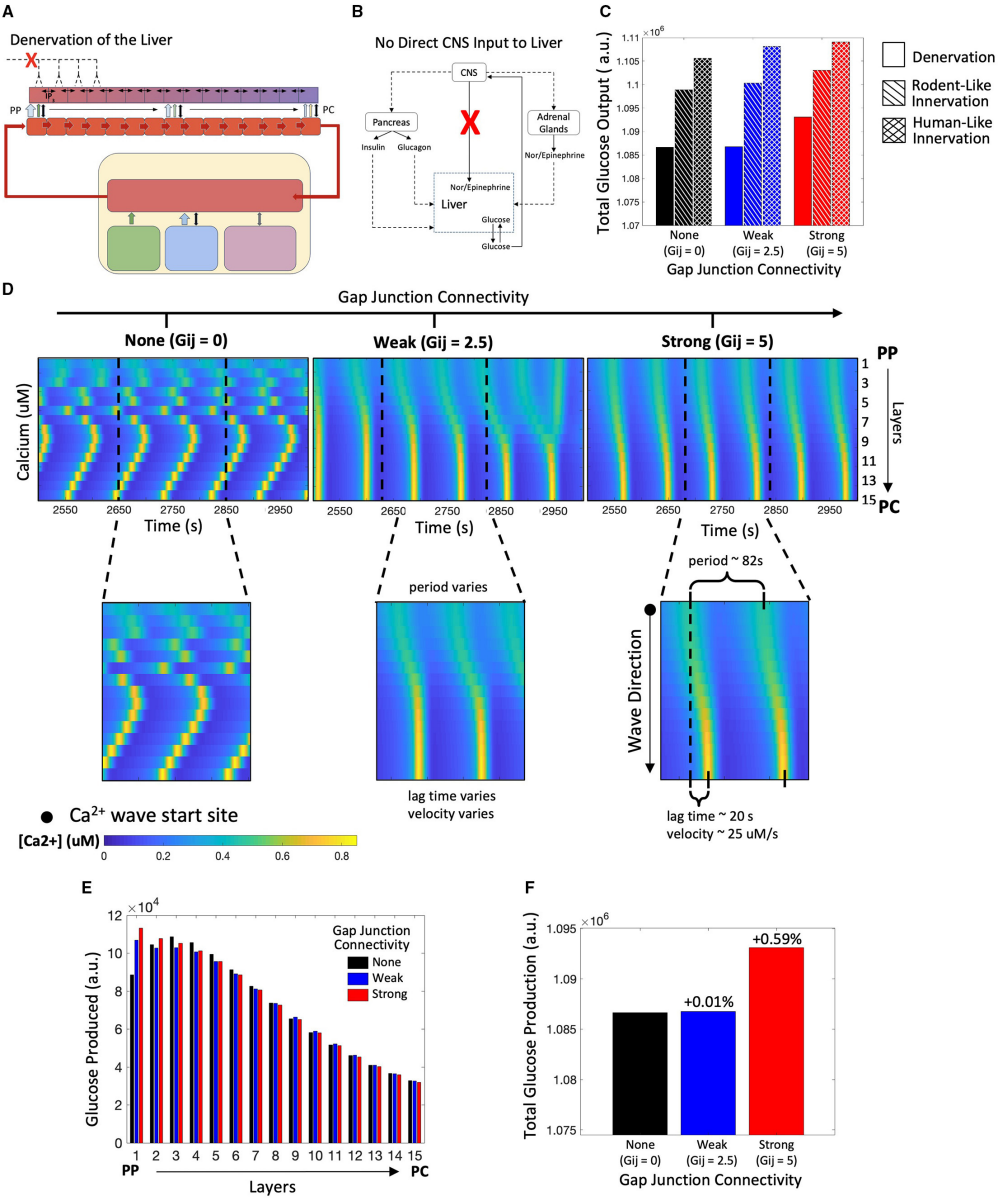
Model of a denervated liver. **(A)** Schematic representation of a denervated liver. **(B)** Network diagram showing no direct CNS input to the liver. **(C)** Hepatic glucose production is reduced in the denervated liver as compared with the innervated liver. **(D)** Calcium spiking is unsynchronized along the lobule when Gij=0. When gap junction connectivity is weak, calcium waves exhibit “islands” of synchronized Ca^2+^ response. In the strongly coupled system, calcium waves begin in the PP region and propagate toward to PC region. **(E)** The zonation of hepatic glycogenolysis is consistent with system of innervation: hepatic glucose output decreases from the PP to PC region. **(F)** Total glucose output is highest when gap junction connectivity is strong.

Next, we explored the overall hepatic glucose response to hormonal signals in the absence of hepatic innervation (Figures 6A,B). We were interested in examining whether gap junction coupling between adjacent hepatocyte layers would provide a mechanism for local tissue scale coordination of calcium signaling in the absence of top-down neural inputs to each hepatocyte layer. We simulated the model to explore the effect of change in gap junction coupling on hepatic glucose output in the case of liver denervation. The denervated state of the liver was represented in our model by excluding the CNS activation dependent additional stimulus received by hepatocytes (*K*_*Stim*_ = 0 in Equation 16).

Our simulations reveal that total glucose output is diminished in the completely denervated liver as compared with the human-like and rodent-like innervated models (Figure 6C). In denervated livers, Ca^2+^ waves start in the PP region and propagate toward the PC region (Figure 6D). In this case, the Ca^2+^ waves follow the gradient of circulating catecholamines across the liver lobule, which is the sole model species regulating Ca^2+^ response in the absence of innervation. An interesting case arises for low gap junction conductivity where a superposition of lobular scale and localized Ca^2+^ responses result in complex signaling patterns (Figure 6D, *t* = 2,950). We have previously reported “islands” of synchronized Ca^2+^ response within lobular scale Ca^2+^ waves in murine livers (Verma et al., 2018). Our simulations indicate that low gap junction connectivity in combination with extracellular stimulus profiles could drive complex Ca^2+^ signaling patterns across liver lobules. The simulated zonation of glycogenolysis in denervated livers is consistent with that in innervated livers, with PP hepatocytes exhibiting the highest levels of glycogenolysis (Figure 6E).

In denervated livers, strong gap junction connectivity can increase hepatic glucose output (Figure 6G). The increase in hepatic glucose output observed in the simulations can be attributed to lobular scale Ca^2+^ signaling. In our model, PP hepatocytes show the highest rate of glycogenolysis but the lowest Ca^2+^ spiking amplitudes in response to a mobilizing stimulus. Strong gap junction connectivity results in a more robust Ca^2+^ spiking in the PP hepatocytes which further increases glycogenolysis rates.

**Figure 7:**
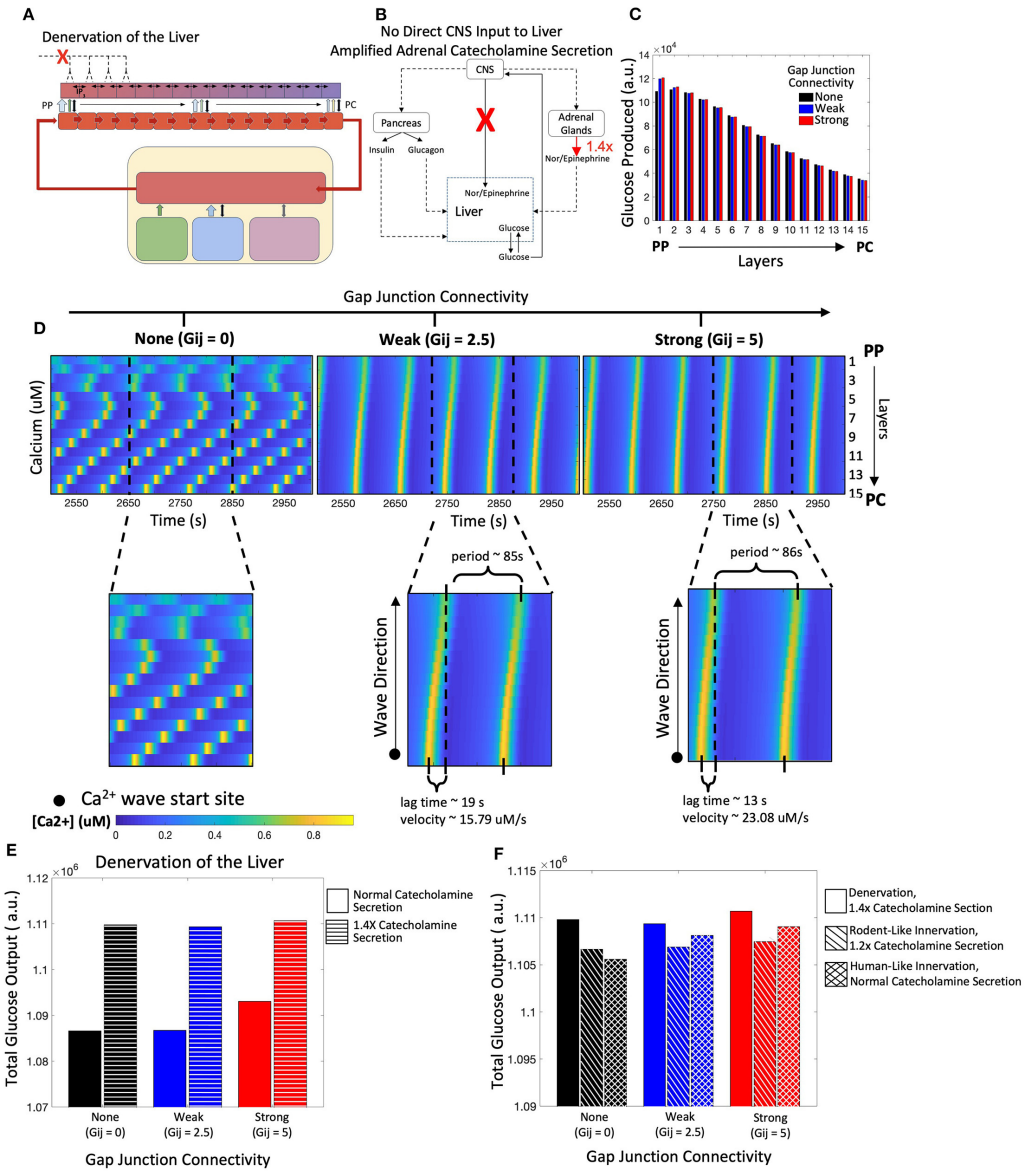
Model of a denervated liver with amplified adrenal catecholamine secretion (1.4X). **(A)** Schematic representation of a denervated liver. **(B)** Network diagram of systemic signals with the adrenal glands increasing catecholamine secretion by 1.4-fold. **(C)** Hepatic glucose production decreases from the PP to PC region, consistent with simulations from the denervated liver with normal catecholamine secretion. **(D)** Calcium waves now propagate from the PC to PP region given weak and strong gap junction connectivity and increased catecholamine secretion. Calcium wave velocities increase with strength of gap junction connectivity. **(E)** There is an increase in total glucose output from a denervated system with normal catecholamine secretion to one with increased catecholamine section (1.4X). **(F)** Total glucose output is consistent across scenarios of denervation with a 1.4-fold increase in catecholamine secretion, rodent-like innervation with a 1.2-fold increase in catecholamine secretion, and human-like innervation with normal catecholamine secretion rates.

In the absence of liver innervation, our model simulations show a PP to PC flow of Ca^2+^ signal in the lobule. However, *ex vivo* experiments have revealed that Ca^2+^ waves propagate from the PC to PP region (Verma et al., 2018). Increasing the base catecholamine synthesis rate by 1.4-fold from the adrenal glands as represented schematically in Figures 7A,B, and restored the experimentally observed directionality of Ca^2+^ signal propagation in our simulations (Figure 7C). In addition, there is an increase in hepatic glucose output compared with simulations of the denervated liver under normal circulating catecholamine levels (Figure 7E). Total glucose output is consistent across simulations of liver denervation with a 1.4-fold increase in rate of catecholamine secretion, rodent-like innervation with a 20% increase in catecholamine secretion, and human-like innervation with baseline rates of catecholamine secretion, all despite varying gap junction connectivity (Figure 7F). Comparing the results in Figures 6, 7 with that of Figures 4, 5, it appears that strong gap junctional connectivity is needed as the hepatic innervation diminishes in order to maintain the calcium spatial patterns elicited by the integrated effect of circulating and synaptically released catecholamines in the liver.

### Modeling the Impact of Portal Hypertension Mediated Increase in Sinusoidal Blood Flow

**Figure 8:**
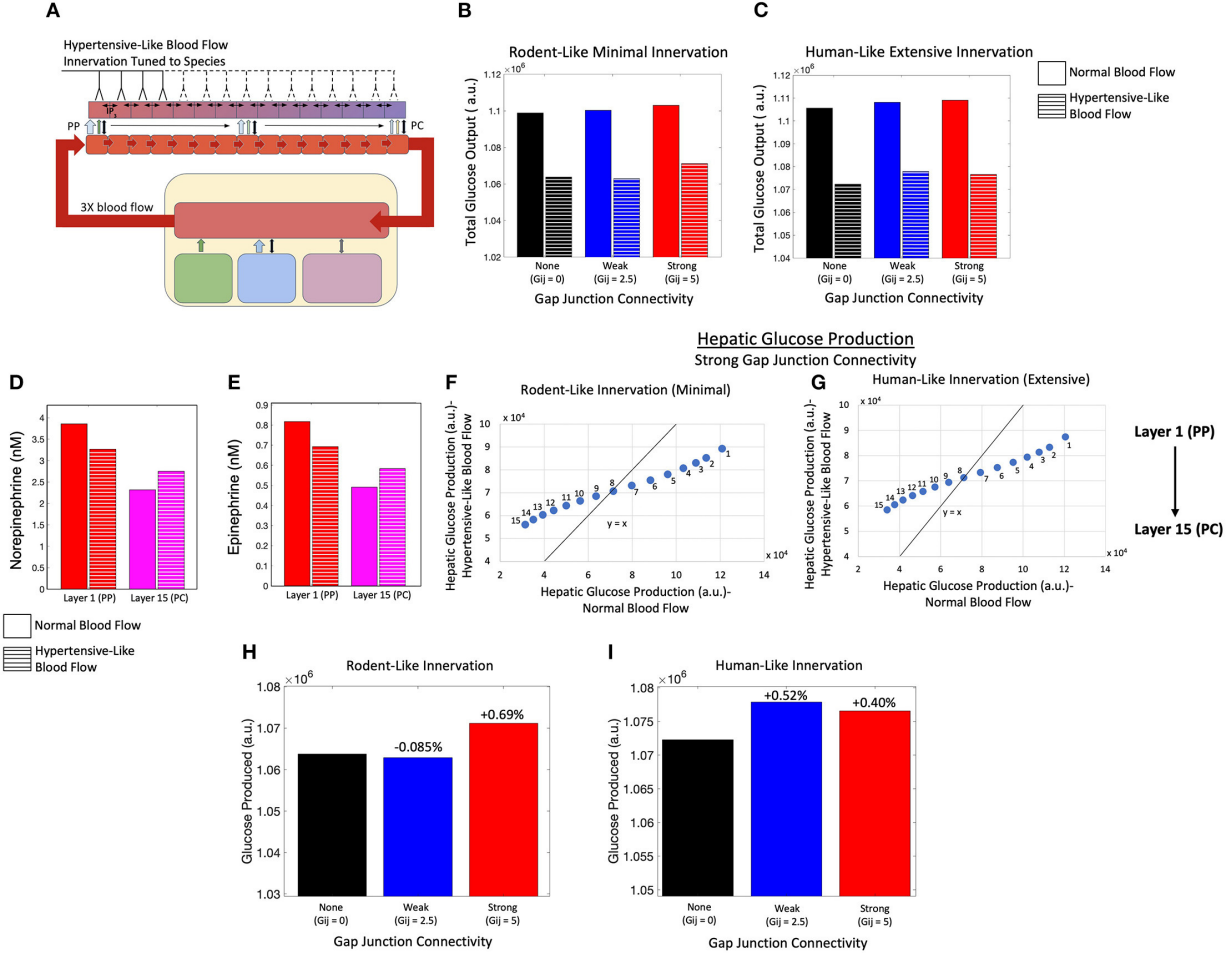
Model of an innervated liver with hypertensive-like blood flow. **(A)** Schematic representation of a liver with hypertensive-like blood flow and innervation tuned to species. **(B)** Total hepatic glucose production is higher in a system of rodent-like innervation with normotensive conditions compared with hypertensive conditions, despite gap junction connectivity. **(C)** Total hepatic glucose production is higher in a system of human-like innervation with normotensive conditions compared with hypertensive conditions, despite gap junction connectivity**. (D)** The hepatic norepinephrine concentration decreases in the PP region and increases in the PC region during hypertension relative to normotensive conditions. **(E)** The hepatic epinephrine concentration also decreases in the PP region and increases in the PC region during hypertension relative to normotensive conditions. **(F)** Given minimal rodent-like innervation, PC (PP) hepatic layers show higher (lower) glucose output in the hypertensive case as compared with the normotensive case. **(G)** In agreement with rodent-like innervation, extensive human-like innervation simulations also show PC (PP) hepatic layers with higher (lower) glucose output in the hypertensive case as compared with the normotensive case. **(H)** Total glucose output was highest when gap junction connectivity was strong in the case of minimal rodent-like innervation. **(I)** Total glucose output was highest when gap junction connectivity was weak in the case of extensive human-like innervation.

Next, we investigated the effect of elevated blood flow rates on hepatic glucose output driven by catecholamines during periods of increased systemic glucose demand (Figure 8A). We used Poiseuille's law to calculate the blood flow rate through liver lobules corresponding to a 3-fold increase in portal pressure (see Methods). An increase in blood flow serves as a proxy for simulating hepatic Ca^2+^ and glycogenolytic dynamics in cases with increase in portal hypertension. Consistent with clinical findings (Joly et al., 1967; Gaudin et al., 1991), we simulated equal rates of catecholamine secretion from the adrenal glands for normotensive and portal hypertensive cases and focused our analysis on the interplay of innervation and blood flow to modulate hepatic glycogenolysis in the two cases.

Simulation results indicate lower hepatic glucose output with increased rate of blood flow, irrespective of the extent of innervation (rodent-like minimal or human-like extensive) and the gap junction connectivity, suggesting that the capacity of the liver to break down intracellular glycogen reserves into glucose is reduced in the case of higher sinusoidal blood flow (Figures 8B,C). The reduction in hepatic glucose output is consistent with reduced catecholamine concentration profiles in the portal compartment (Figures 8D,E). Systemic catecholamines also experience a decrease in concentration during portal hypertensive cases as the glucose synthesized by hepatocytes enter the systemic compartment faster during periods of increased blood flow. This is reflected by a lower drop in systemic glucose during portal hypertension, as displayed in Supplementary Figure 3A. In addition, the simulated hemodynamic changes lead to a redistribution of circulating catecholamines across the lobule resulting in altered glycolysis rates throughout the lobule. Increase in blood flow rates results in a shallower gradient of circulating catecholamines (Supplementary Figures 3B,C). The effect of catecholamine redistribution through the lobule is reflected in the simulated zonal contribution of hepatocytes toward total hepatic glucose output. In rodent-like and human-like innervation schemes, hepatocytes closer to the PC (PP) region showed higher (lower) glucose output in the hypertensive case compared with the normotensive case, as indicated by the PP hepatocytes lying below and PC hepatocytes lying above the diagonal line (Figures 8F,G).

Interestingly, our simulations show that intercellular communication and extent of innervation played an important role in hepatic glucose response to hormonal/neural signals in the case of elevated sinusoidal blood flow. For rodent-like innervation, hepatic glucose output was highest for strong gap junction connectivity in normotensive as well as hypertensive cases (Figure 8H). However, for human-like innervation with portal hypertension, hepatic glucose output is highest when gap junction connectivity is weak (Figure 8I). Our simulations thus show that in addition to zonation and Ca^2+^ signaling, blood flow could add as an additional factor regulating lobular patterns of glycogenolysis.

Due to redistribution of circulating catecholamines discussed previously, Ca^2+^ response is dampened in the portal hypertensive case. Increased intercellular communication (i.e., stronger gap junction connectivity) led to an improved intracellular Ca^2+^ response in the PP hepatocytes (Supplementary Figures 4A,B) and consequently, to a net increase in hepatic glucose output compared with the weakly coupled system given rodent-like minimal innervation (Figure 8H). In a system with human-like extensive innervation, however, weak gap junction connectivity resulted in the highest total glucose output due to a higher overall Ca^2+^ signal resulting in minimal increases in midlobular and PC glucose production relative to the system with strong gap junction connectivity (Figure 8I; Supplementary Figures 4C,D).

## Discussion

In this work, we presented a systematic analysis of the regulation of Ca^2+^ signal propagation and hepatic glucose production by zonation, multi-organ interactions, and cell-cell communication in different modes of hepatic innervation. Starting with a previously developed model of hepatic glucose metabolism (Ashworth et al., 2016), we added the effects of CNS activation on the liver directly through innervation and indirectly through increased stimulation of the adrenal glands to release systemic hormones. We expanded the model further to include 15 hepatic layers, and incorporated calcium signaling to capture the effect of gap-junction mediated Ca^2+^ responses on hepatic glycogenolysis. Our simulations show that hepatic glycogenolysis is regulated primarily by glucagon and that catecholamine induced Ca^2+^ dynamics result in only small increases. In all simulated cases, there is a PP to PC zonation of glycogenolysis within liver lobules. However, lobular scale Ca^2+^ dynamics are different despite the initialization of the cell-intrinsic Ca^2+^ signaling parameters being the same across all simulated cases. Variability in spatio-temporal Ca^2+^ dynamics in liver lobules can cause small perturbations in glycogenolysis rates in different hepatocyte layers.

Our simulations demonstrate that the complex interplay of intracellular and multi-organ interactions can override the default modes of Ca^2+^ dynamics and spatial distribution of glycogenolysis in liver lobules. It has been experimentally shown that cell intrinsic Ca^2+^ signaling components are overexpressed in the PC region compared with the PP region. This leads to propagation of Ca^2+^ waves from the PC region to the PP region in gap junction coupled hepatocytes. However, our simulations indicate that depending on the extent of innervation and circulating catecholamines, Ca^2+^ waves can start at different points in lobules showing canonical zonation patterns. Although glycogenolysis is consistently higher in the PP region in all the simulated cases, innervation, gap junction connectivity and circulating catecholamines can influence total hepatic glucose production across liver lobules differentially. Additionally, our simulations show that increased gap junction connectivity resulted in increased hepatic glucose synthesis.

We note that across many simulated cases, the difference in magnitude of total hepatic glucose output is small. One of the key factors likely responsible for the small differences is initialization of cell-intrinsic metabolic and Ca^2+^ signaling parameters. Cell-intrinsic parameters are held constant across all simulations, with the same values used in Ashworth et al. (2016) and Verma et al. (2016), for ease of interpretation of simulation results. However, we expect there to be substantial inter-subject variability, and possibly distinct zonation profiles across liver lobules in these parameters. Nevertheless, our analysis provides qualitative insights into the trends of shifts in intra-hepatic processes that can be expected when modulating key processes. High-resolution proteomic data acquired for model calibration using novel high throughput techniques such as matrix assisted laser desorption ionization (MALDI) will enable more precise quantification of these differences.

Gap junction-mediated synchronization of Ca^2+^ signal propagation has been investigated experimentally in the context of cholestasis, chronic ethanol adaptation, and, more recently, hepatic metabolism (Kruglov et al., 2011; Bartlett et al., 2017; Gaspers et al., 2019, 2021). Our simulations provide novel predictions regarding catecholamine-induced hepatic glycogenolysis in portal hypertension and the role of gap junctions in hypertensive human subjects. Our simulations indicate lower glycogenolysis in highly innervated livers during periods of increased systemic glucose demand under hypertension. Under normal blood flow rates, increase in gap junction coupling leads to increased hepatic glucose output in highly innervated livers. In contrast, hepatic glucose output from highly innervated livers with portal hypertensive blood flow is the highest under weak gap junction coupling. These results suggest that hepatic blood flow is an additional regulatory component that can modulate hepatic glycogenolysis.

Our simulations show that stimulation of adrenal glands by the CNS and the resulting increase of systemic catecholamines can reverse the suppression of hepatic glycogenolysis caused by loss of liver innervation. This is an interesting case in which the site of catecholamine release (adrenal glands) is distinct from the organ of interest (the liver) and is mediated by a third remote entity (CNS), capable of reversing glycogenolytic effects. Novel targeted therapies, rooted in peripheral organ stimulation of the heart (Hanna et al., 2021), gut (Ma et al., 2019), and stomach (Tan et al., 2021) have gained increasing attention recently. The present results using multi-scale modeling of complex interactions between multiple organs across different spatial and temporal scales can support future studies to develop and test neurostimulation therapies. This work and similar efforts could provide a computational proof-of-concept platform for assessing feasibility of such therapies, in addition to providing useful mechanistic insight into the underlying phenomena.

In this research, we combined a model of hepatic energy metabolism (Ashworth et al., 2016) with a model of lobular scale Ca^2+^ signaling (Verma et al., 2016) and added multi-organ interactions to explore the zonal patterns of hepatic glycogenolysis across liver lobules. Similar strategies could be used to explore interesting extensions of our study. For example, the integrated model considers a one-dimensional liver lobule wherein each hepatocyte has either one or two adjacent hepatocytes. Extension of the model to 2-D, 2.5-D, or 3-D cases would be capable of describing a more realistic case with multiple cell-cell interactions between hepatocytes and their effect on overall hepatic glycogenolysis. Availability of specialized applications such as CompuCell3D (Swat et al., 2012) could facilitate building and simulations of spatially accurate computational models. The scope of the model can be further increased by integrating larger models of hepatic physiology such as HepatoNet (Gille et al., 2010). The model can also be extended for computational investigation of disease pathologies such as the interaction of endoplasmic reticulum (ER) stress and hepatic insulin resistance. The mechanisms of obesity associated ER stress and its role in hepatic insulin resistance are well-documented (Kim et al., 2015; Villalobos-Labra et al., 2019). Additionally, ER stress could impact intracellular Ca^2+^ signaling adversely (Krebs et al., 2015). Inclusion of these relevant mechanisms into the current model could provide further insight into the lobular scale Ca^2+^ dynamics and energy metabolism in liver lobules. The extent to which insulin resistance can be reversed by therapeutic strategies targeting intra-hepatic and systemic signals and processes can then be elucidated.

## Data Availability Statement

The original contributions presented in the study are included in the article/Supplementary Material, further inquiries can be directed to the corresponding author/s.

## Author Contributions

AV, AM, and RV designed the study. AV and AM developed the model. AM and RN performed simulations. JH, BO, and RV supervised simulations. AV, AM, JH, BO, and RV interpreted the results. AV, AM, RV, and BO edited the manuscript. All authors contributed to the article and approved the submitted version.

## Funding

This work was financially supported by the National Institute of Biomedical Imaging and Bioengineering U01 EB023224 and National Institute on Alcohol Abuse and Alcoholism R01 AA018873. The funding sponsors had no role in the design of the study, in the collection, analyses, or interpretation of data, in the writing of the manuscript and in the decision to publish the results.

## Conflict of Interest

The authors declare that the research was conducted in the absence of any commercial or financial relationships that could be construed as a potential conflict of interest.

## Publisher's Note

All claims expressed in this article are solely those of the authors and do not necessarily represent those of their affiliated organizations, or those of the publisher, the editors and the reviewers. Any product that may be evaluated in this article, or claim that may be made by its manufacturer, is not guaranteed or endorsed by the publisher.

## Supplementary Material

The model file in XPP format can be found in Supplementary File 1 (ModelFile.ode). The alternate model in XPP format can be found in Supplementary File 2 (AlternateModelFile.ode). The MATLAB file used for running the simulations on the XPP-MATLAB interface can be found in Supplementary File 3 (RunOdeFiles.m). The document describing the self-assessed conformance of our model to the state of the art Ten Simple Rules for Credible Practice in Modeling and Simulation in Healthcare can be found in Supplementary File 4.
