# Supplementary material for: A Spatial Model of Hepatic Calcium Signaling and Glucose Metabolism Under Autonomic Control Reveals Functional Consequences of Varying Liver Innervation Patterns Across Species
Source: Front Physiol. 2021 Nov 26;12:748962. doi: 10.3389/fphys.2021.748962 (PMC8662697; doi:10.3389/fphys.2021.748962)
Supplement: Supplementary file 1 [file Data_Sheet_1.DOCX]

Supplementary Material

# Supplementary Files

**Supplementary File 1:** Modelfile.ode contains the code for implementing the model in XPP.

**Supplementary File 2:** AlternateModelfile.ode contains the code for implementing the alternate model in XPP.

**Supplementary File 3:** RunOdeFiles.m contains the code for running the model using Matlab and XPP-Matlab interface.

**Supplementary File 4:** Self-assessment of the conformance of the present study to the Ten Simple Rules for Credible Practice of Modeling and Simulation in Healthcare.

#
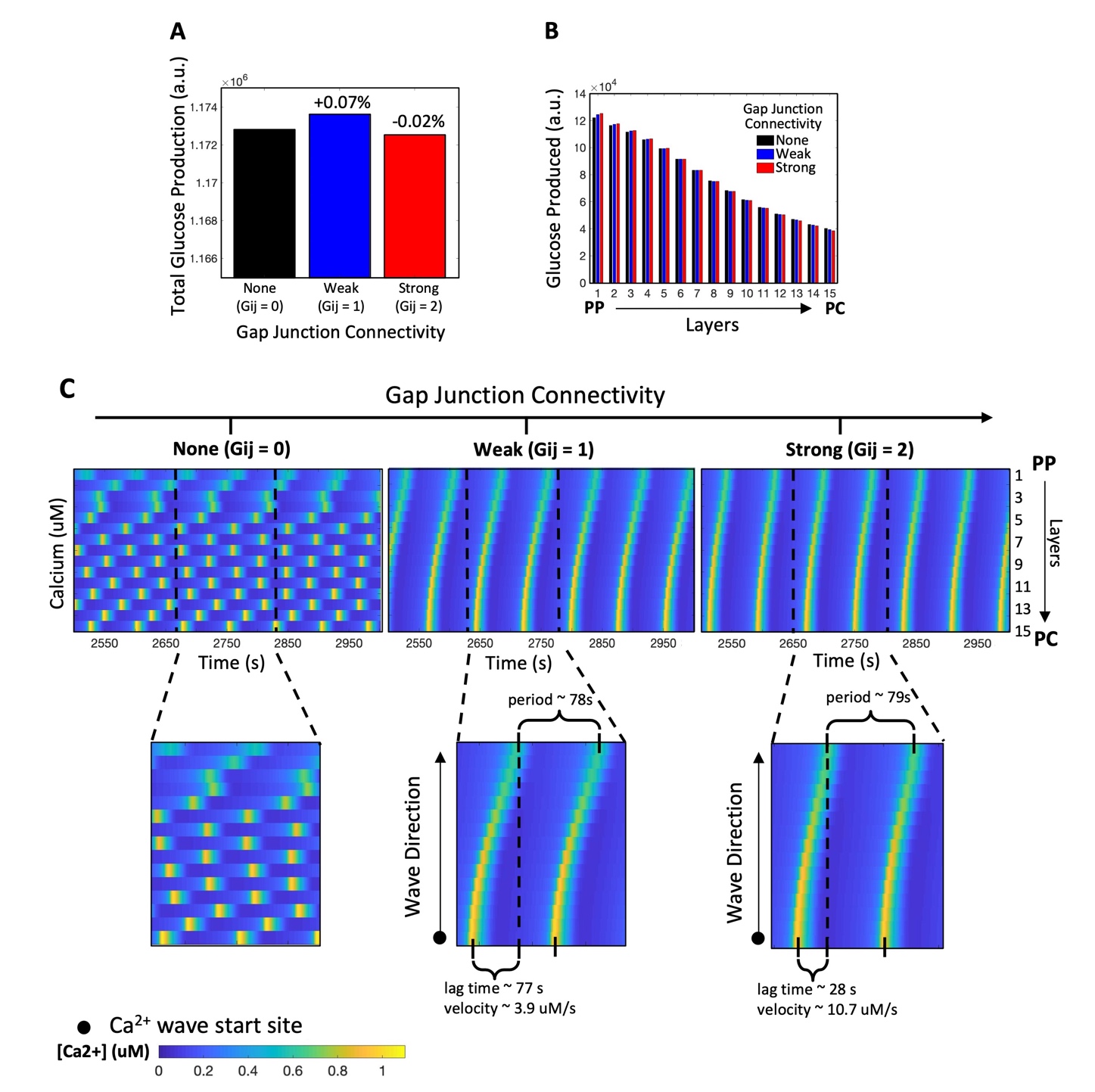
Supplementary Figures

**Supplementary Figure 1.** Model alternative. The simulations show key model readouts for human-like innervation. The simulation strategy is as described in the **Methods** (Section 2.13: Model Alternative). (**A**) Hepatic glucose output in the simulated case is the highest for weak gap junction connectivity (vs. strong gap junction connectivity in the main model). (**B**) PP zonation of glycogenolysis is preserved, consistent with results from the main model. (**C**) Lobular scale Ca^2+^ signaling patterns are similar to the main model for human-like extensive innervation.


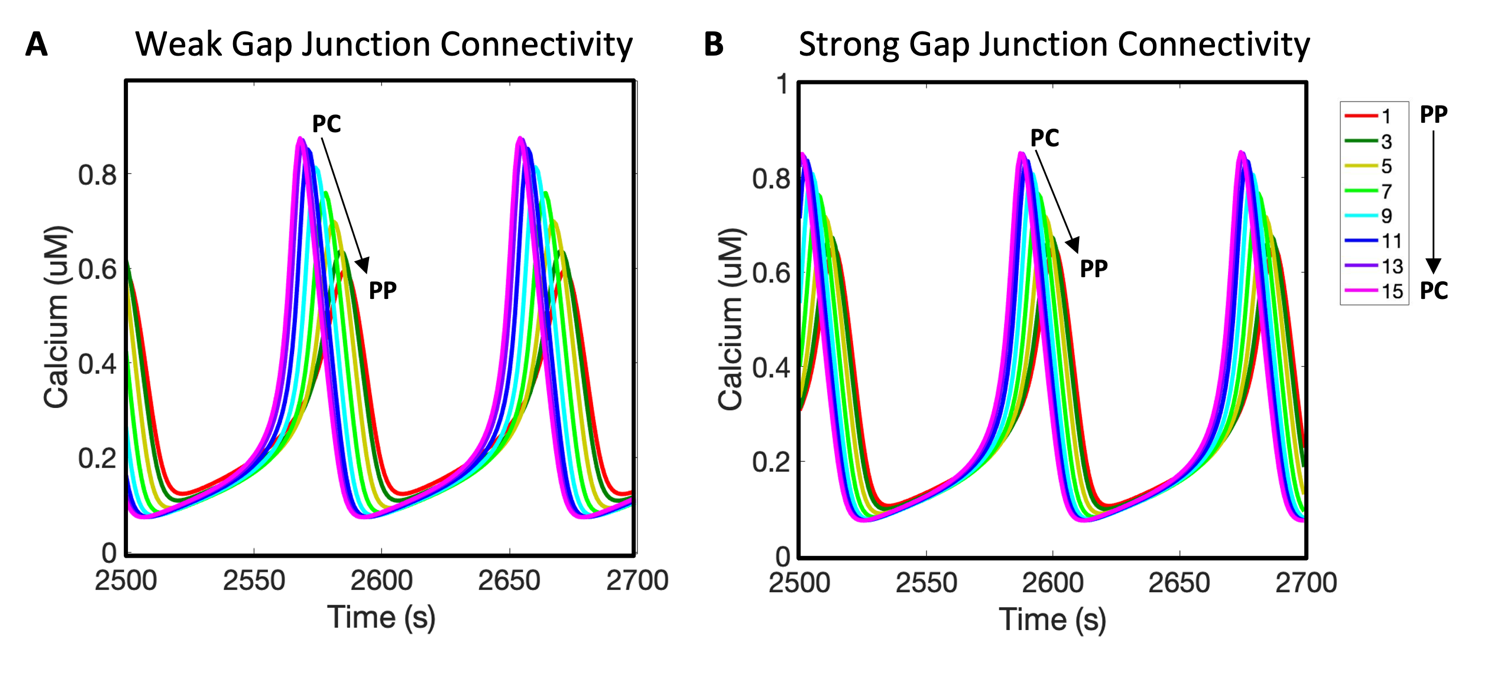
**Supplementary Figure 2.** Calcium spiking decreases in amplitude from the PC to PP region for human-like extensive innervation simulations. **(A)** Weak gap junction connectivity (Gij = 2.5). **(B)** Strong gap junction connectivity (Gij = 5).

**
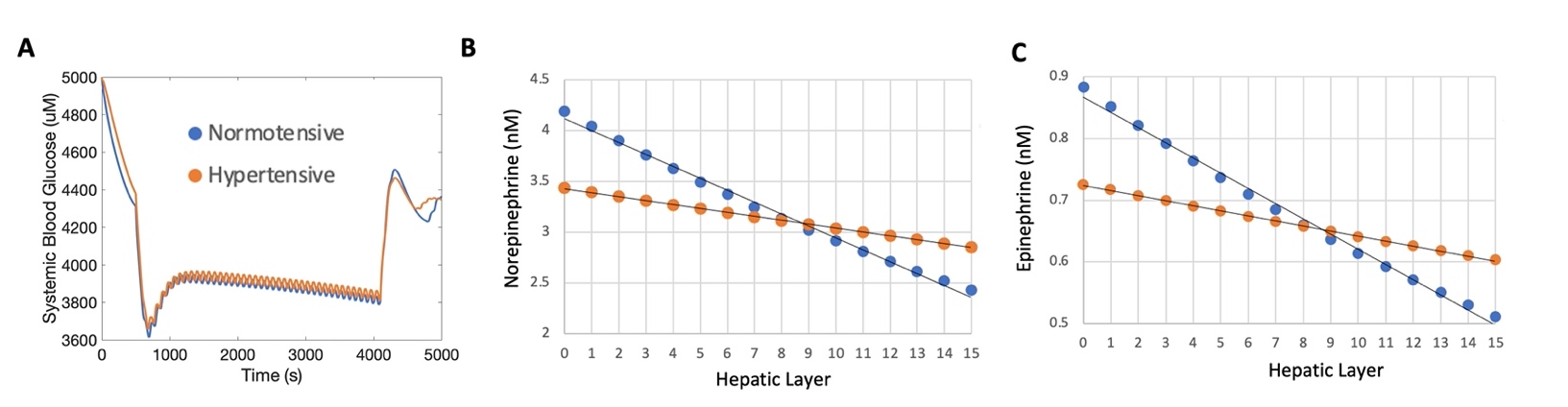
**

**Supplementary Figure 3.** Changes in systemic glucose and catecholamine concentrations during hypertension relative to normotensive conditions given human-like extensive innervation. **(A)** Systemic blood glucose levels drop lower during normotensive conditions compared to hypertensive conditions. A shallower gradient of norepinephrine (**B**) and epinephrine (**C**) exists from the systemic to PC region during hypertensive conditions. 0 = systemic compartment in (**B**) and (**C**).

**
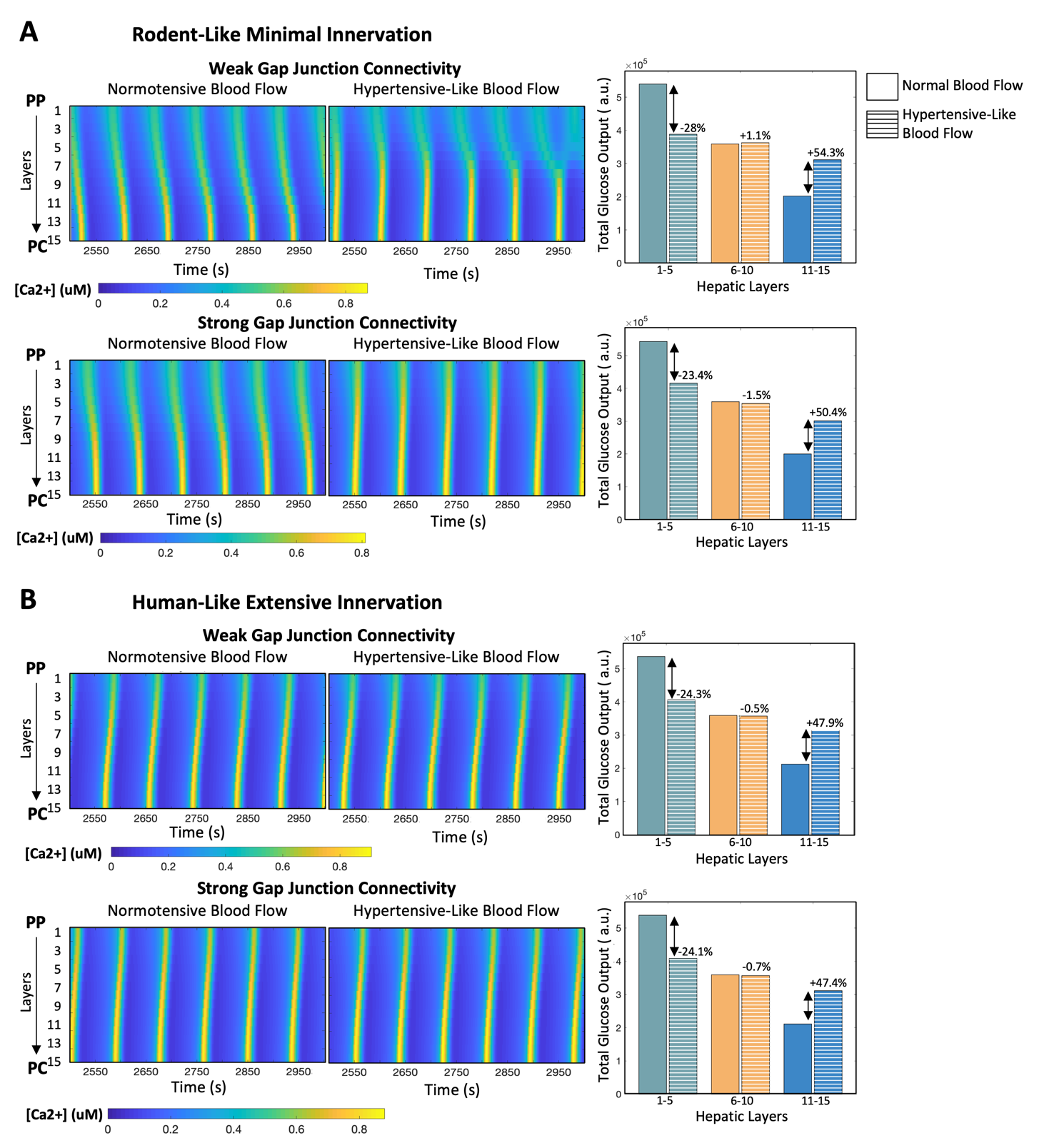
­­­­Supplementary Figure 4.** Hypertensive Ca^2+^ dynamics and regional hepatic glucose output given varying extents of innervation and gap junction connectivity. **(A)** Simulations of rodent-like minimal innervation under hypertension show an improved Ca^2+^response and an increase in hepatic PP glucose production when gap junction connectivity is strong. **(B**) Simulations of human-like extensive innervation under hypertension showed a stronger Ca^2+^ response and an increase in hepatic midlobular and PC glucose production when gap junction connectivity is weak.
