# Supplementary material for: A Spatial Model of Hepatic Calcium Signaling and Glucose Metabolism Under Autonomic Control Reveals Functional Consequences of Varying Liver Innervation Patterns Across Species
Source: Front Physiol. 2021 Nov 26;12:748962. doi: 10.3389/fphys.2021.748962 (PMC8662697; doi:10.3389/fphys.2021.748962)
Supplement: Supplementary file 5 [file Data_Sheet_2.docx]

**Self-assessment of conformance to the Ten Simple Rules of Credible Practice in Modeling and Simulation in Healthcare**

A Spatial Model of Hepatic Calcium Signaling and Glucose Metabolism under Autonomic Control Reveals Functional Consequences of Varying Liver Innervation Patterns Across Species

The following self-assessment is based on the rules specified in Erdemir et al. (2020) and the rubric available at: <https://www.imagwiki.nibib.nih.gov/content/10-simple-rules-conformance-rubric>

Date of initial self-assessment: July 28, 2021

Date of second self-assessment upon manuscript revision: September 20, 2021

**Rule 1: Define context clearly:** Develop and document the subject, purpose, and intended use(s) of the model or simulation.

**Current Conformance Level:** Comprehensive

**Model Context:** Model glycogenolysis and calcium signaling in a multiscale multiorgan model with varying extent of hepatic innervation.

**Primary goal of the model/tool/database:** The primary objective of the modeling study was to evaluate the role of neural signals in controlling the metabolic functionality of liver, particularly in regulating the glycogenolysis to maintain appropriate response to hormonal signals to meet the systemic glucose demands.

Our model builds on a previously developed model of hepatic glucose metabolism. We extended this model by integrating calcium signaling and direct and indirect control of liver metabolism by the central nervous system through catecholamines. This newly developed model with spatial organization and multi-scale features can be utilized to explore intercellular and multi-organ interactions governing calcium signaling in liver lobules and hepatic glycogenolysis. Our expanded multi-scale, multi-organ model of hepatic metabolism incorporates intracellular metabolism, liver zonation, lobular scale calcium signaling by systemic hormones, hepatic innervation, and direct and peripheral organ-mediated communication between the liver and the central nervous system. Simulations can be performed to compare regulation of liver glucose metabolism across as well as within species. In addition, the model can be simulated to examine the influence of innervation and gap junction connectivity on hepatic glucose output.

**Biological Domain of the Model:** Glucose metabolism, calcium signaling, and autonomic control

**Structures of the Model**: Liver, central nervous system (CNS), adrenal glands, pancreas, blood

**Spatial Scales Included in the Model:** intercellular (1 to 20 um), lobular (10 um to 300 um), systemic/organ (cm to m)

**Time Scales Included in the Model:** 0 to 5000 seconds

**Other uses for the model (optional):** The model also includes aspects of lipid metabolism, whose parameterization can be altered and explored further to mimic a hepatic disease state. The effect of dietary intake and insulin resistance in promoting a hepatic steatosis-like phenotype can be explored in the context of innervation, calcium signaling, and CNS activation.

**Additional comments about the model’s context (optional)**: The model was assessed under a fasted organismal state with increased systemic glucose demand. These conditions should be considered when applying to future work.

**Revision summary:**

This stayed consistent during the revision period.

**Rule 2: Use contextually appropriate data:** Employ relevant and traceable information in the development or operation of a model or simulation.

**Current Conformance Level:** Extensive

| **Data for building the model** | **Published?** | **Private?** | **How is credibility checked?** | **Current Conformance Level** |
| --- | --- | --- | --- | --- |
| in vitro (primary cells cell, lines, etc.) | Yes | No | the source data is confirmed to meet detailed data requirements for consistency and source description | Extensive |
| ex vivo (excised tissues) | Yes | No | the source data is confirmed to meet detailed data requirements for consistency and source description | Extensive |
| in vivo pre-clinical (lower-level organism or small animal) | Yes | No | the source data is confirmed to meet detailed data requirements for consistency and source description | Extensive |
| in vivo pre-clinical (large animal) | N/A | N/A | N/A | N/A |
| Human subjects/clinical | Yes | No | the source data is confirmed to meet detailed data requirements for consistency and source description | Extensive |

**Revision summary:**

This stayed consistent during the revision period.

| **Data for validating the model** | **Published?** | **Private?** | **How is credibility checked?** | **Current Conformance Level** |
| --- | --- | --- | --- | --- |
| in vitro (primary cells cell, lines, etc.) | N/A | N/A | N/A | N/A |
| ex vivo (excised tissues) | N/A | N/A | N/A | N/A |
| in vivo pre-clinical (lower-level organism or small animal) | Yes | No | the source data is confirmed to meet detailed data requirements for consistency and source description | Adequate |
| in vivo pre-clinical (large animal) |  |  |  |  |
| Human subjects/clinical | Yes | No | the source data is confirmed to meet detailed data requirements for consistency and source description | Adequate |

**Revision summary:**

This stayed consistent during the revision period.

**Rule 3: Evaluate within context:** Perform verification, validation, uncertainty quantification, and sensitivity analysis of the model or simulation with respect to the reality of interest and intended use(s) of the model or simulation.

**Current Conformance Level:** Extensive

|  | **Who Does It?** | **When does it happen?** | **How is it done?** | **Current Conformance Level** |
| --- | --- | --- | --- | --- |
| **Verification** | Developer | During development | Comparison of model output with published animal data | Extensive |
| **Validation** | Lab Member | During development | model was used to reproduce simulations and figures | Extensive |
| **Uncertainty Quantification** | User performs uncertainty quantification | Can be performed every time the model is run for a new scenario | User discretion | Adequate |
| **Sensitivity Analysis** | User performs sensitivity analysis on influential parameters | Can be performed after every new simulation | User discretion | Adequate |

**Revision summary:**

Extensive validation was performed during the revision process. The model was recalibrated based on experimental hepatic calcium dynamics and catecholamine secretion in humans during periods of increased exercise.

**Rule 4: List limitations explicitly:** Provide restrictions, constraints, or qualifications for or on the use of the model or simulation for consideration by the users or customers of a model or simulation.

**Current Conformance Level:** Comprehensive

| **Disclaimer statement (explain key limitations)** | **Who needs to know about this disclaimer?** | **How is this disclaimer shared with that audience?** | **Current Conformance Level** |
| --- | --- | --- | --- |
| Limited human/rodent kinetic data for parameterization | Users | Stated explicitly in the main text | Comprehensive |
| Parameterization of the model is the same for human and rodent-like simulations, only differing by extent of innervation | Users | Stated explicitly in the main text | Comprehensive |
| Parameterization of the model is the same for hypertensive scenario, only differing by rate of blood flow | Users | Stated explicitly in the main text | Comprehensive |
| Small changes in total glucose output across simulation scenarios leads to mostly qualitative assessment of trends | Users | Stated explicitly in the main text | Comprehensive |

**Revision summary:**

This stayed consistent during the revision period.

**Rule 5:** **Use version control:** Implement a system to trace the time history of modeling and simulation activities including delineation of each contributors’ efforts.

**Current Conformance Level:** Extensive

|  | **Naming Conventions?** | **Repository?** | **Code Review?** |
| --- | --- | --- | --- |
| **individual modeler** | N/A | Github | Yes |
| **within the lab** | Yes | Yes | Yes |
| **collaborators** | N/A | Github | Yes |

**Revision summary:**

Version 2 of the code, including both the main and alternative models, can be found on GitHub (<https://github.com/Daniel-Baugh-Institute/SpatialLiverModel>; ver. 2, 2021).

**Rule 6:** **Document appropriately:** Maintain up-to-date informative records of all modeling and simulation activities, including simulation code, model mark-up, scope and intended use of modeling and simulation activities, as well as users’ and developers’ guides.

**Current Conformance Level:** Extensive

|  | **Current Conformance Level** |
| --- | --- |
| **Code Commented?** | Extensive: comments made in the model file |
| **Scope and intended use described?** | Extensive: described in the main text |
| **User’s Guide** | Extensive: described in the main text and supplemental files |
| **Developer’s Guide?** | Partial: Details of model development in methods of main text |

**Revision summary:**

Model alternative and revisions are explained in the main text. Supplemental Figure 1 shows the results from the previous model version (now considered as a Model Alternative).

**Rule 7: Disseminate broadly:** Share all components of modeling and simulation activities, including simulation software, models, simulation scenarios and results.

**Current Conformance Level:** Extensive

| **Target Audience(s):** | **“Inner Circle”** | **Scientific Community** | **Public** |
| --- | --- | --- | --- |
| **Simulations** |  |  | Description of simulations stated in the main text |
| **Models** |  |  | Model file present in supplementary material and on GitHub. |
| **Software** |  |  | MATLAB, XPP and XPP-MATLAB interface were used. All of these are publicly available either freely or for a fee. |
| **Results** |  |  | Described in main text |
| **Implication of Results** |  |  | Described in main text |

**Revision summary:**

Version 2 of the model is now available on Github.

**Rule 8: Get independent reviews**: Have the modeling and simulation activity reviewed by nonpartisan third-party users and developers.

**Current Conformance Level:** Extensive

| **Reviewer(s) name and affiliation** | **Alison Moss (Thomas Jefferson University)** |
| --- | --- |
| When was the review performed | July 19, 2021 |
| How was review performed and outcomes of the review? | A member of the research group, not involved in the present study and does not conduct research in liver biology, performed the review.  Model files and tables in the text were cross-checked for consistency.  Simulation results and figures were independently reproduced using the files provided on Github. |

**Revision summary:**

This stayed consistent during the revision period.

**Rule 9: Test competing implementations**: Use contrasting modeling and simulation implementation strategies to check the conclusions of different strategies against each other.

**Current Conformance Level:** Adequate

|  | **Yes or No (briefly summarize)** |
| --- | --- |
| **Were competing implementations tested?** | Yes, in multiple stages.  Competing implementations were tested and compared by the first three authors of the paper during the initial manuscript preparation.  During the manuscript revision, the model was revised further, labeling the initial model as a Model Alternative. |
| **Did this lead to model refinement or improvement?** | Yes, in both stages.  The initial model was refined and improved whenever inconsistencies arose. Specifically, final model was extended from 8 layers in a liver lobule in the initial implementation to contain 15 layers. A new simulation was added to account for changes in the blood flow due to portal hypertension. Parameters for gap junctions were altered to yield physiologically consistent calcium dynamics.  During the manuscript revision, the model was recalibrated to account for experimental patterns of circulating catecholamines and calcium signals. |

**Revision summary:**

Updated the text to include model revisions and alternatives.

**Rule 10: Conform to standards:** Adopt and promote generally applicable and discipline specific operating procedures, guidelines, and regulations accepted as best practices.

**Current Conformance Level:** Adequate

|  | **Yes or No (briefly summarize)** |
| --- | --- |
| **Are there operating procedures, guidelines, or standards for this type of multiscale modeling?** | Yes, as described in the credible practice of modeling and simulation in healthcare: ten rules from a multidisciplinary perspective (Erdemir et al., 2020). |
| **How do your modeling efforts conform?** | Our model is implemented in the widely used Matlab platform for computational modeling. We also used another freely available and popular software, XPP, along with its Matlab interface. The code is commented at critical locations to aid the reader. |

**Revision summary:**

This stayed consistent during the revision period.

**References:**

Erdemir, A., Mulugeta, L., Ku, J. P., Drach, A., Horner, M., Morrison, T. M., Peng, G., Vadigepalli, R., Lytton, W. W., & Myers, J. G., Jr (2020). Credible practice of modeling and simulation in healthcare: ten rules from a multidisciplinary perspective. Journal of translational medicine, 18(1), 369. https://doi.org/10.1186/s12967-020-02540-4
